# Genome-wide amplification of proviral sequences reveals new polymorphic HERV-K(HML-2) proviruses in humans and chimpanzees that are absent from genome assemblies

**DOI:** 10.1186/s12977-015-0162-8

**Published:** 2015-04-28

**Authors:** Catriona M Macfarlane, Richard M Badge

**Affiliations:** Department of Genetics, University of Leicester, University Road, Leicester, LE1 7RH UK

**Keywords:** Endogenous retrovirus, HERV-K, HML-2, Provirus, Human genome

## Abstract

**Background:**

To date, the human population census of proviruses of the *Betaretrovirus*–like human endogenous retroviral (HERV-K) (HML-2) family has been compiled from a limited number of complete genomes, making it certain that rare polymorphic loci are under-represented and are yet to be described.

**Results:**

Here we describe a suppression PCR-based method called genome-wide amplification of proviral sequences (GAPS) that selectively amplifies DNA fragments containing the termini of HERV-K(HML-2) proviral sequences and their flanking genomic sequences. We analysed the HERV-K(HML-2) proviral content of 101 unrelated humans, 4 common chimpanzees and three centre d’etude du polymorphisme humain (CEPH) pedigrees (44 individuals). The technique isolated HERV-K(HML-2) proviruses that had integrated in the genomes of the great apes throughout their divergence and included evolutionarily young elements still unfixed for presence/absence.

**Conclusions:**

By examining the HERV-K(HML-2) proviral content of 145 humans we detected a new insertionally polymorphic Type I HERV-K(HML-2) provirus. We also observed provirus versus solo long terminal repeat (LTR) polymorphism within humans at a previously unreported, but ancient, locus. Finally, we report two novel chimpanzee specific proviruses, one of which is dimorphic for a provirus versus solo LTR. Thus GAPS enables the isolation of uncharacterised HERV-K(HML-2) proviral sequences and provides a direct means to assess inter-individual genetic variation associated with HERV-K(HML-2) proviruses.

**Electronic supplementary material:**

The online version of this article (doi:10.1186/s12977-015-0162-8) contains supplementary material, which is available to authorized users.

## Background

Endogenous retroviruses (ERVs) are relics of exogenous retroviral infections of germ cells that result in integration of proviral DNA into the host genome [1]. Throughout primate evolution HERV families have integrated into germline DNA and subsequently been vertically transmitted as Mendelian traits [2]. Proviral insertions may be subject to host selection or be selectively neutral; in either case they will ultimately be fixed or lost from the population, such that presence/absence polymorphism is usually transient [3]. Over time, most HERV proviruses become replication defective through the accumulation of mutations in their coding sequences. Alternatively, homologous recombination between directly orientated LTRs that flank the provirus results in deletion of internal coding sequence and the formation of a solo LTR [4]. Approximately 8% of the human genome is composed of retrovirus-like sequences [5], with solo LTRs being tenfold more abundant than proviruses [6].

Proviral sequences of the HERV-K(HML-2) family are the best preserved and most biologically active, with some having maintained the ability to produce functional proteins and retrovirus-like particles [3,7-14]. Although molecular reconstruction of a putative HERV-K(HML-2) progenitor demonstrates that the family could be weakly capable of re-infection [15,16], a naturally occurring replication-competent provirus has yet to be identified.

HERV-K(HML-2) viruses first integrated into the germline of an Old World primate ancestor 30 to 35 million years ago, following their divergence from New World primates [17]. The oldest proviral loci, HERV-K(OLD), are characterised by longer *gag* and LTR sequences and proliferated within primates until separation of the lineage leading to gorilla [18]. Their proviral descendants, Type I and Type II, are distinguished by a 292 bp deletion at the *pol-env* boundary and appeared prior to the branching of the lineage leading to orangutan [18,19]. Type I genomes harbour the deletion and are incapable of independent extracellular re-infection as they cannot encode a functional Env protein [20]. Type I and Type II proviruses have amplified equally within the human lineage following divergence from chimpanzee [4,21], with five proviruses being polymorphic for presence/absence in humans [22-25].

Initial sequencing of human and chimpanzee genomes revealed that the HERV-K(HML-2) family was represented by 73 human specific insertions (63 solo LTRs and 7 proviruses) [5] and 45 chimpanzee specific insertions (44 solo LTRs and 1 provirus) [26]. This data was interpreted to indicate increased activity in the human lineage following speciation, with expansion estimated to have taken place in the hominin germline between 1.5 and 4 mya [27-29].

HERV-K(HML-2) integrants have been detected by a variety of methods including hybridisation screening of genomic libraries [20,22,30,31], bioinformatic analyses of reference genome assemblies [4,5,18,21,23-26,28,29,32-41] and suppression PCR with, and without, subtractive hybridisation [42-46].

Although highly informative, these studies have examined the proviral content of a small number of individuals or haplotypes and so rare low allele frequency insertions will be under-represented [41,47]. Indeed, under a model of current or very recent activity it is expected that rare HERV-K(HML-2) insertions may exist that have escaped detection [24]. Furthermore, the under-representation of insertionally polymorphic HERV-K(HML-2) loci in the human reference genome has been demonstrated by comparison with archaic hominin genomes [38,39,48].

Here we describe GAPS, which selectively amplifies DNA fragments containing the termini of HERV-K(HML-2) proviruses and their flanking sequences. The method initially targets the proviral gene *gag* or *env,* and then enriches for sequences containing an adjacent LTR; as a result detection is restricted to near complete and full-length proviruses. The assay systematically avoids amplification of HERV-K(HML-2) solo LTRs and the HERV-K10 LTR-containing SINE-R VNTR-Alu (SVA) retrotransposons, which are numerous within chimpanzee and human genomes [49,50]. Our results demonstrate the recovery of previously undescribed HERV-K(HML-2) proviral sequences which are absent from reference genome assemblies. Based upon recovery of 10 of the 26 now known human specific HERV-K(HML-2) proviruses, we expect to be able to recover around 38% of new insertions. GAPS is a simple high-throughput PCR-based method to directly assess inter-individual variation in HERV-K(HML-2) integrants which are polymorphic for insertion of a provirus or provirus versus solo LTR.

## Results

### Genome-wide amplification of HERV-K(HML-2) proviral sequences (GAPS)

**Figure 1:**
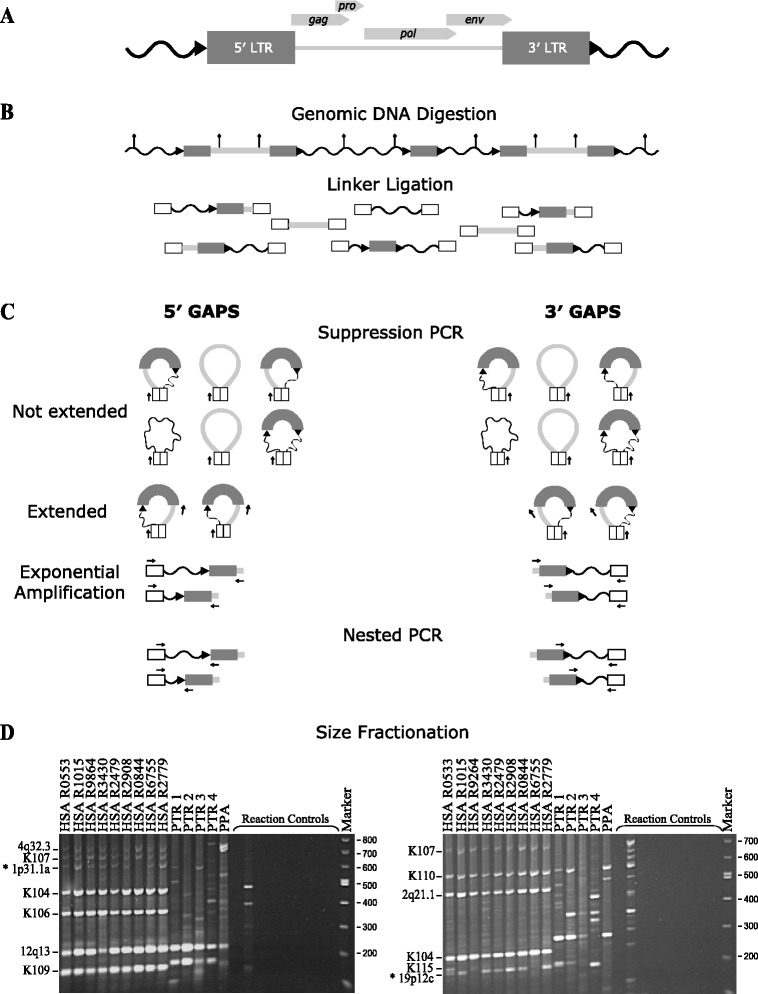
Schematic representation of GAPS and size fractionation of amplicons from humans and chimpanzees. **(A)** HERV-K(HML-2) provirus containing viral genic regions *gag, pro, pol* and *env* bordered by two LTRs, direct repeats () and unique flanking DNA (). **(B)** Linkered genomic DNA library generation. Restriction sites () within a representative section of genomic DNA that contains two proviruses and a solo LTR is shown at the top. Following digestion annealed linkers (white boxes) are ligated to genomic DNA restriction fragments, which then serve as templates for PCR. **(C)** GAPS PCR amplifications. 5′ GAPS targets the 5′ end, and 3′ GAPS the 3′ end of proviral sequences. Arrows () indicate primer binding sites. Following suppression PCR melting, self-complementary linkers form duplexes creating a population of stem-looped DNA molecules. Extension from the linker primer is prevented as primer annealing is blocked by linker duplex formation at the base of the stem. Molecules containing the binding site for the target primer of choice (GAG or ENV) are extended and become templates for exponential amplification. Nested PCR is performed to enrich for sequences containing a LTR and reduce amplicon length. **(D)** Size fractionation by gel electrophoresis. 5′ GAPS on left and 3′ GAPS on right. Genomes include nine humans (HSA) and four chimpanzees (PTR). Lane 14 (PPA) is a positive control utilising bonobo DNA. The remaining controls were for construction of linker-genomic DNA fragments and were set up using the same genomic DNA sample as Lane 9. Library controls (left to right): No genomic DNA, no restriction enzyme, no linker ligated genomic DNA, no ligase and no linker. The final four controls were PCR negatives with DNA omitted. Numbers on the left are DNA size marker fragments (bp). Loci retrieved using GAPS are indicated on the right. New polymorphic proviruses discovered using GAPS (*). Refer to Additional file 1 for the un-cropped gel image.

We modified amplification typing of L1 active subfamilies (ATLAS) [51,52] to selectively amplify HERV-K(HML-2) proviral sequences from oligonucleotide-linkered genomic libraries in a method termed GAPS (Figure 1). Genomic DNA from three CEPH pedigrees (44 individuals), 101 unrelated human (ECACC Ethnic Diversity DNA panel) and four unrelated chimpanzee genomes was digested to completion with the restriction enzyme *Vsp* I, generating restriction fragments (Figure 1B). The digested DNA was ligated to GC-rich double stranded linkers and suppression PCR was performed using a proviral gene region specific primer (GAG for 5′ GAPS and ENV for 3′ GAPS) and a linker specific primer (Figure 1C). During suppression PCR intra-molecular annealing of sections of genomic DNA that have linkers attached at both ends leads to the formation of stable stem-loop structures that suppress PCR amplification. The suppression effect is relieved if a target primer (specific for proviral *gag* or *env*, Figure 1C) anneals within the loop of the panhandle structure and is extended, creating an amplicon containing a single linker sequence, which amplifies exponentially. A second nested PCR reaction was performed to enrich for DNA fragments containing 5′ or 3′ proviral LTR-junction fragments and reduce the size of the GAPS amplicons. The resultant multiple amplicons were size fractionated on agarose gels and visualised by ethidium bromide (EtBr) staining (Figure 1D and Additional files 1 and 2). Amplicons of interest were excised from the gel and directly sequenced.

### Isolation of HERV-K(HML-2) proviral sequences from genomic DNA

**Figure 2:**
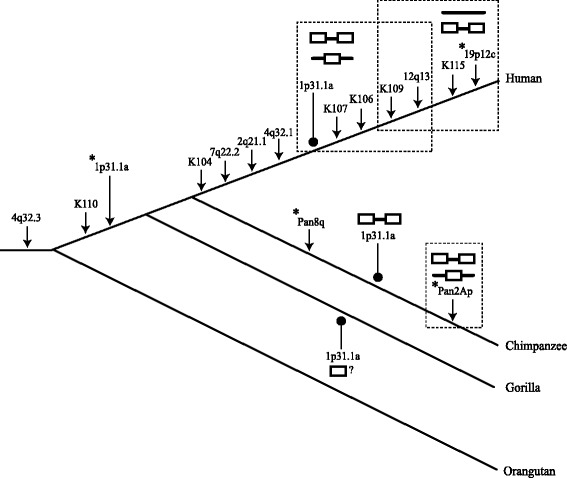
Cladogram of the great apes overlaid with insertions and structural variations of GAPS acquired loci. Estimated times of insertion of each HERV-K(HML-2) GAPS loci () is illustrated within the phylogeny of human, chimpanzee and gorilla. HERV-K(HML-2) loci contained within dashed boxes are dimorphic for the structural variants shown.  = Pre-insertion site.  = Provirus.  = Solo LTR.  = LTR present but locus structure is unknown. The allelic variants of the 1p31.1a locus observed within each species are indicated (). New polymorphic loci discovered using GAPS are highlighted with asterisks (*).

To examine HERV-K(HML-2) proviral complement within humans and chimpanzees, GAPS was performed on genomic DNA samples from 101 unrelated humans, 4 unrelated common chimpanzees (*Pan troglodytes*) and three CEPH pedigrees (n = 44). Resolution of 5′ GAPS and 3′ GAPS products of 14 individuals is shown in Figure 1D (see Additional file 1 for full resolution Figure 1D gels and Additional file 2 for data from the ECACC Ethnic Diversity DNA panel and CEPH pedigree amplicons). In total, 18 different proviral 5′ and 3′ LTR junction fragments, of various sizes, were recovered. Comparison of their flanking regions revealed that they represented 15 distinct HERV-K(HML-2) loci. All 15 loci were confirmed present in the relevant reference genome, using the UCSC and NCBI Genome Browsers. Ten loci were human specific, two were chimpanzee specific and the remaining three loci occurred in both species (Figure 2 and Additional file 3). The HERV-K proviruses were categorised as subtype Type I or Type II, depending upon the absence of a 292 bp segment spanning the *pol-env* gene boundary (Type I) or its presence (Type II). We retrieved ten Type I and three Type II proviruses. The remaining two insertions could not be classified as Type I or Type II due to absence of the diagnostic region or because these loci are represented only by solo LTR sequences in the reference genome. Analysis of these insertions’ LTR subtype by RepeatMasker [53] showed that both were LTR5Hs, with one being LTR subtype Hs-a and the other Hs-b [54].

All of the human specific loci revealed by GAPS were previously reported [14,20,22,24,31,37,55]. However, one proviral locus, named 19p12 [14], showed dimorphism within the resolved GAPS amplicons, which had not been previously reported. The proviral 3′ flank-LTR junction sequence was also absent from the reference human (hg19) and archaic hominin genomes [Denisovan: UCSC Read: M_SOLEXA-GA03_00031_PEdi_MM_SR_1:1:119:7088:5616 #AATCTTC,NCGCAGG and Neanderthal Vi33.25: UCSC Read: C_M_SOLEXA-GA03_JK_PE_SL32:3:27:1079:211]. Further BLAST searches with the proviral LTR-junction fragment within the NCBI nucleotide database and human trace archives confirmed that this locus was polymorphic with respect to insertion. The provirus was present within accession [GenBank: AC008996] and the pre-insertion site was present in accession [GenBank: AC073539], both of which were mapped to chromosome region 19p12 (Table 1). As the centromeric region 19p12 in humans contains several human specific HERV-K(HML-2) loci, including the insertionally polymorphic provirus K113, this provirus was allocated the unique name 19p12c. We suggest that two of the four HERV-K(HML-2) proviruses located at 19p12 be renamed (Additional file 4) and refer to these proviruses as such herein. Sequence alignment of the proviral 19p12c sequence to the sequence HERV-K10 [GenBank: M14123] [20] indicated that the 19p12c provirus lacked a 5′ LTR and was a Type I proviral genome.

**Table 1:** **Characteristics of new HERV-K(HML-2) proviral loci**

**Locus** ^**a**^	**Location**	**Type**	**Direct repeat**	**Allele frequency**			**Inter-LTR divergence**	**Estimate of proviral integration (mya)**
				**PI**	**Provirus**	**sLTR**		
*19p12c*	19p12	I	NA^b^	0.57	0.43	0.0	NA^b^	NA^b^
*Pan8q*	8q	I	AGGAGA	0.0	1	0.0	0.008	2.42 (1.57-3.27)
*Pan2Ap*	2Ap	U^c^	GCTG(G\A)TGGGCGAGAA(AAA/---)	0.0	1	0.0	0.006	1.81 (1.17-2.45)
*1p31.1a*	1p31.1	I	CATGT	0.0	0.965	0.035	(HSA)^d^ 0.004	1.205 (0.78-1.63)
							(PTR) 0.026	7.87 (5.09-10.65)

^a^Newly identified HERV-K loci were named according to their chromosome band location in humans or chromosome arm in chimpanzee. PI = Pre-Insertion site allele. ^b^NA = Not applicable as one of the proviral LTRs is absent. ^c^U = Undetermined as the reference genome assembly contains a solo LTR. ^d^HSA = Human sequence. PTR = Chimpanzee sequence.

GAPS analysis of four chimpanzee genomic DNAs revealed two chimpanzee specific proviral loci, which were named Pan8q and Pan2Ap (Figure 2). Pan8q was positioned on chromosome 8 with an intact pre-insertion site sequence being present in the gorilla reference genome (gorGor3) (centered on position chr8: 113581183) and also in human accession [GenBank: AC064802] (Table 1 and Additional file 3). The provirus was full-length with the *gag* ORF being interrupted by an insertion of 1338 bp of a HERV-K(HML-2) LTR - *gag* sequence, indicative of either a duplication or integration/deletion event following insertion of the full-length provirus. Flanking the LTRs were identical 6 bp direct repeats, and the proviral genome was of Type I subtype. The second novel proviral locus, Pan2Ap, was located on chromosome 2a within the chimp reference genome assembly and was represented by a solo LTR. Unusually long degenerate direct repeats of 15/18 bp (Table 1) surrounded this solo LTR and further sequencing of the Pan2Ap LTRs from DNA sample PPT1, demonstrated the same 15/18 bp direct repeats also flanked the proviral allele (refer to submitted sequence accessions). An uninterrupted pre-insertion site for Pan2Ap was present within the gorilla reference genome gorGor3 (centered on position chr2a: 9239200) and in the human accession [GenBank: AC112723]. As GAPS is designed to isolate proviral sequences and the chimp genome assembly contained a solo LTR at this locus, these results indicated allelism for a solo LTR versus a provirus, at the chimpanzee specific Pan2Ap locus (Figure 2).

Two of the three loci that were shared between the humans and chimpanzees (K110 and 4q32.3), were previously identified [31,33]. In chimpanzee, a long and a short GAPS product variably represented the 4q32.3 locus. Sequence analysis revealed that this variation was due to a SNP generating a new *Vsp* I restriction enzyme site (Additional file 5), rather than insertional or structural polymorphism.

The third shared proviral locus, 1p31.1a, had not been previously reported in humans or chimpanzees. 1p31.1a is located on the short arm of chromosome 1 in human, with the chimp reference genome containing a provirus and the human reference genome a solo LTR in accession [GenBank: AL606535] (Table 1) (Additional files 3 and 6). As a distinct human specific HERV-K(HML-2) provirus called 1p31.1 (also known as K116 and ERVK-1), which has variable alleles for a solo LTR and provirus, has previously been observed to be located at the same cytogenetic location [23] we named the new locus 1p31.1a. We suggest that 1p31.1 (K116) be renamed 1p31.1b (Additional file 4) and refer to this provirus as such hereafter. The chimpanzee provirus and human solo LTR alleles of 1p31.1a were flanked by identical direct repeats of 5 bp, and the chimpanzee proviral genome was of the Type I subtype (Table 1).

Examination of the next generation sequencing (NGS) data of the Denisovan genome sequence [UCSC Read: M_SOLEXA-GA03_00031_PEdi_MM_SR_1:8:55:8960:13295#ACCAACG,ATGGAGA] and Neanderthal Vi33.16 genome sequence [UCSC Read: M_SL-XAQ_0002_FC30R0F AAXX:7:92:1338:1611] displayed on the UCSC Genome Browser indicated that 1p31.1a was also present in these genomes (Additional file 6). However the sequences covering the flank-HERV-K(HML-2) junction were insufficient to determine allelic structure. Further BLAT searches of the primate reference genomes showed that the 1p31.1a locus was also present as a 526 bp 3′ truncated LTR within the gorilla (gorGor3) and absent from the orangutan (PonAbe2) (Additional file 3). Under the assumption that HERVs are identical by descent markers and cannot be removed without deletion of the surrounding flanking sequences [56], the presence of 1p31.1a in human, chimpanzee and gorilla indicates that it integrated within a common ancestor, around the time of or following the evolutionary separation of the orangutan lineage (Figure 2).

A proviral allele of 1p31.1a was isolated by GAPS and the human genome reference assembly contained a solo LTR, indicating dimorphism for a solo LTR versus provirus, in an ancient HERV-K(HML-2) insertion within human populations (Figure 2). This allelism was further confirmed by its presence in the database of genomic variation (DGV) [57], where it corresponds to variation 33955.

### Genotyping of novel proviral loci

**Figure 3:**
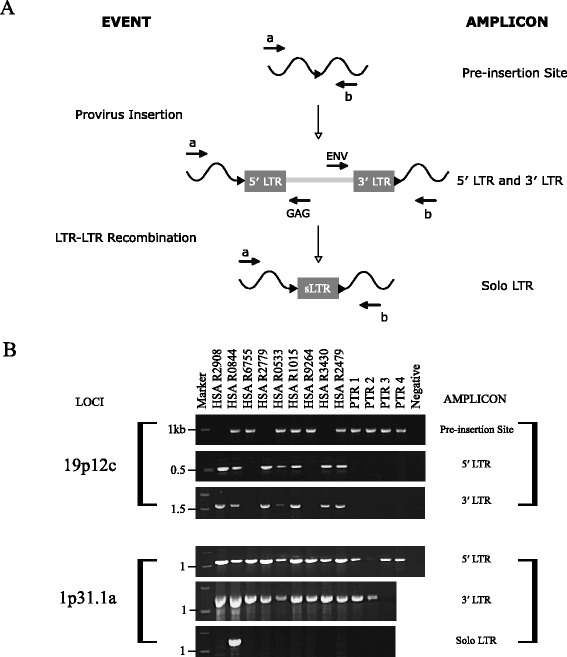
Detection of structural variation at HERV-K(HML-2) loci. **(A)** Formation of alleles and strategy for independent amplification of structural variations in novel human HERV-K(HML-2) proviral loci. Primer binding sites are indicated by arrows (). The locus specific primer combination a + b amplifies the pre-insertion site and solo LTR. Primer combination a + GAG amplifies the 5′ LTR of a full-length provirus and ENV + b the 3′ LTR. **(B)** Composite agarose gels of locus specific amplification for allelic variation. Numbers on the left correspond to the co-migrating DNA size marker (kb). Samples are the same as used in Figure 1D, with humans in a different order. The identity of each band was confirmed by DNA sequencing. Refer to Additional file 7 for un-cropped gel images.

To confirm the species specificity and polymorphism of the HERV-K(HML-2) proviral loci 19p12c, Pan2Ap, Pan8q and 1p31.1a, PCR primers were designed based on their unique flanking sequences. Human and common chimpanzee genomic DNA samples were genotyped by PCR amplification for the absence of the provirus, as evidenced by a short uninterrupted genomic target sequence (pre-insertion site), or the presence of a larger amplicon containing a solo LTR. Two independent amplifications were used to characterise the proviral allele using primer combinations specific for the peripheral proviral LTRs (Figure 3A). With the exception of the Pan2Ap locus, PCR analysis confirmed the allelism indicated by GAPS and *in silico* analysis of the genome reference assemblies (Figure 3B and Additional files 7, 8 and 9). The provirus 19p12c was verified as human specific and dimorphic for insertion with a proviral allele frequency of 0.57 in the 101 unrelated, ethnically diverse individuals tested. Pan2Ap and Pan8q were confirmed to be species specific, with all four common chimpanzee individuals carrying proviral alleles. In agreement with the GAPS assay, 1p31.1a was demonstrated to be a full-length provirus, present in both humans and chimpanzees. Finally, congruent with the human genome reference assembly (hg19), several individuals also possessed a solo LTR at this locus, with an allele frequency of 0.035 (n = 101).

### Phylogenetic analysis of GAPS loci

**Figure 4:**
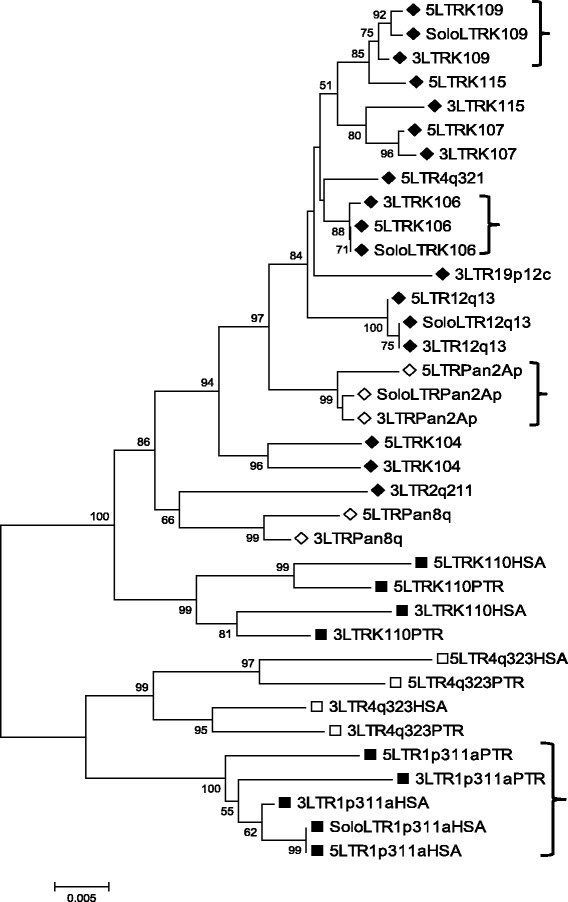
Phylogeny of HERV-K(HML-2) LTRs. Neighbor-joining tree of LTRs from loci recovered using GAPS. Bootstrap values over 50% are shown. Individual LTRs are named according to the chromosomal location or the bibliographic name of the locus. 5′ and 3′ LTRs are indicated by 5 and 3. Solo LTRs by Solo. ♦ = Human specific. ◊ = Chimp specific. ■ = Present in human, chimp and gorilla. □ = Present in human, chimp, gorilla and orangutan. HSA = human copy of LTR. PTR = chimp copy of LTR. Parentheses (}) highlight proviral loci that are also represented by a solo LTR.

The phylogenetic relationships of LTRs of the proviral loci detected using GAPS were examined (Figure 4). A neighbour-joining tree formed a comb-shaped topology similar to those obtained previously for HERV-K sequences [3,4,27,33]. As the 5′ and 3′ LTRs of a provirus are identical at the time of retroviral integration and subsequently evolve independently of each other, they provide a source of phylogenetic signal, which in the absence of sequence exchange, may serve as a molecular clock [56,58]. With the exception of the K115 proviral locus, all 5′ and 3′ proviral LTRs grouped as sister taxa with bootstrap support of > 70% (500 replicates), signifying that each provirus inserted into the germline as a result of an independent infection event. The anomaly of the K115 proviral LTRs may be due to gene conversion between the LTRs [22]. This insertion is within the distal repeat (REPD) copy number variable beta-defensin region where gene conversion is ongoing (reviewed in [59]).

The solo LTRs for the species specific loci K106, K109, 12q13 and Pan2Ap all grouped with their respective 5′ and 3′ proviral LTRs (Figure 4). This observation is consistent with the model that solo LTR formation, via homologous recombination between the LTRs of a provirus, occurs quickly after insertion [4,24,60,61]. In striking contrast the ancient 1p31.1a insertion integrated into the common ancestor of human, chimpanzee and gorilla, but is dimorphic for a solo LTR and provirus within human (Figures 2 and 3) despite 8.5-12 million years of evolution. In addition the 1p31.1a solo LTR also grouped with the human 1p31.1a proviral LTRs, implying that it was recently generated, but the human 1p31.1a proviral LTRs cluster together instead of with their orthologs in chimpanzee (Figure 4). This pattern of similarity is suggestive of intra-LTR sequence exchange in one or both of the orthologous proviruses following species divergence. Similarly, the estimated divergence of the chimpanzee 1p31.1a proviral LTRs is 6.5 times greater than observed in human (Table 1), suggestive of sequence homogenisation between 1p31.1a proviral LTRs in the human lineage.

### Sequence variation within and surrounding the 1p31.1a proviral locus

**Figure 5:**
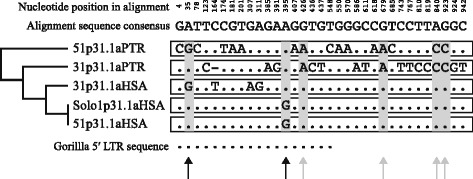
Variable nucleotide sites in a 476 bp alignment of orthologous HERV-K(HML-2) 1p31.1a LTRs. A consensus sequence is shown at the top. Topology of the 1p31.1a LTRs within the phylogeny of GAPS loci is shown on the right. Singleton substitutions have a white background and parsimoniously informative sites are shaded in grey. Sequence of the 526 bp truncated 5′ LTR within gorilla is shown at the bottom. Black arrows show parsimonious differences in the human lineage and grey arrows highlight parsimonious species differences.

To explore the processes that generated a solo LTR at the ancient proviral insertion 1p31.1a, many millions of years after germline integration and fixation, the variable nucleotide sites of the orthologous 1p31.1a LTRs were compared (Figure 5). Within the 976 bp alignment 33 sites were variable, of which 27 were singleton intra-LTR substitutions in either the human or chimpanzee copies of the 1p31.1a provirus. Twenty-four nucleotide differences were present between the chimpanzee 5′ and 3′ LTRs. The human proviral LTRs were distinguished by three variants at positions 164, 307 and 311. Notably the human 1p31.1a solo LTR and 5′ proviral LTR were identical in sequence, indicating that recombination events that generated the solo LTR took place within recent human evolution and perhaps show that the 5′ LTR sequence was conserved during formation of the solo LTR.

Six sites were parsimoniously informative and are highlighted by arrows in Figure 5. Four of these (positions 426, 679, 840 and 923) were different between species, and as the 5′ and 3′ LTRs within the same species have the same nucleotides at each of the sites, indicate sequence homogenisation or gene conversion. The 5′ and 3′ LTRs of a provirus evolve independently of each other following integration [56], so it is unlikely that identical substitutions in both LTRs would arise by chance at four different nucleotides (grey arrows), following the divergence of human and chimpanzee. This is best exemplified by position 426 where the human and gorilla LTRs have a guanine (G) and the chimpanzee LTRs have an adenine (A). The final two informative variations were at positions 35 and 395. Position 35 shows a shared nucleotide between the proviral human 3′ LTR and chimpanzee 5′ LTR and position 395 has a shared substitution between the human solo and proviral 5′ LTR, observations which both support intra-LTR sequence exchange, following species divergence.

It has been reported that the genomic distribution and persistence of full-length proviruses is influenced by gene density and local recombination rate, respectively [62]. We examined the gene density and local recombination rate of the flanking regions surrounding the 1p31.1a locus and the downstream (2.24 Mb) human specific 1p31.1b provirus (Additional file 10). Both of these insertions are dimorphic for a solo LTR and a full-length provirus. Genomic regions 1 Mb upstream and 1 Mb downstream of each of the HERV-K loci were inspected using the UCSC genes track and the deCODE local recombination rate track of Kong et al., [63]. The 1p31 region within which both proviruses reside has been shown to be a region of low gene density when compared to the whole of chromosome 1 [64]. Notably chromosome 1 has an overall density of 14.2 genes per Mb which is almost twice the genome average of 7.8 genes per Mb [64]. Here within a 2 Mb window, we observed 1p31.1a to be flanked by three genes, and 1p31.1b by fourteen known genes including *SLC44A5* (Solute carrier family 55, member 5) within which the provirus is situated (Additional file 10). Low gene density has been implicated in the accelerated fixation of full-length proviruses, perhaps as they are less likely to have a detrimental effect on host gene function [62].

Local recombination rates surrounding each locus were very low: 1p31.1a, 0.1 cM/Mb and 1p31.1b, 0.7 cM/Mb (Additional file 10). It has been shown that full-length proviruses situated in regions of low recombination are less likely to undergo intra LTR-LTR recombination-mediated deletion (generating a solo LTR) and so persist in the full-length state for longer periods of time [62]. However, the effect of local recombination rate in leading to the replacement of a full length provirus by a solo LTR is small in relation to the effect of the mutational divergence of the proviral LTRs [61]. The rapid decline in sequence similarity driven by neutral divergence between LTRs would predict that solo-LTR formation would be an early event in proviral evolution, rather than after a long period of segregation. As previously highlighted in Figure 5 and Table 1, the 5′ and 3′ LTRs of the human orthologue of the 1p31.1a provirus are highly similar. Congruently the downstream 1p31.1 provirus has been observed to have no nucleotide differences between its LTRs [4] and as such is likely to be one of the youngest full-length endogenous retroviruses in the human genome [65]. In view of this, the high intra-LTR sequence similarity of the 1p31.1a and 1p31.1b proviruses, rather than the local recombination rate, is likely to have enabled recombinational deletion of proviral sequence leading to formation of a solo LTR at both of these loci, but at very different times in their evolution.

## Discussion

Application of GAPS to 145 human and four common chimpanzee DNA samples led to the identification of fifteen HERV-K(HML-2) proviruses, ten human specific, two chimpanzee specific and three shared between the two species. Eleven of the proviruses were full length and four were near full length, missing only part of, or one, of their LTRs. Eleven of the proviruses were previously described in an inclusive *in silico* survey that reported 62 HERV-K(HML-2) proviruses with > 87% identity to the polymorphic K113 provirus [37]. As such our *in vitro* method retrieved 17% of HERV-K(HML-2) proviruses previously known to be retained in the human genome.

Notably we identified two HERV-K(HML-2) proviruses in humans which had not been discovered by purely bioinformatic approaches. Thus, we retrieved 20% (13/64) of the total of now known proviruses in the human genome. The first novel provirus, 1p31.1a, integrated and became fixed before diversification of the lineage leading to gorilla, and showed allelism for a solo LTR and provirus in humans. The second provirus, 19p12c, was a Type I provirus polymorphic for insertion in humans. Interestingly the 19p12c provirus was originally discovered through mapping of RNA transcripts found in the plasma of HIV-1 infected individuals [14] showing this provirus is actively transcribed.

In chimpanzees we identified two previously unreported species-specific HERV-K(HML-2) proviruses. The first, Pan8q, was present in the reference genome and confirmed the continued proliferation of Type I proviruses in chimpanzees around the time of or following the split from the human lineage. A full-length Type I provirus (HERV-K GC1) has previously been identified in chimpanzees and is absent from humans [26,28,36]. However HERV-K GC1 is also present in bonobo and gorilla genomes demonstrating that it entered the germline prior to speciation of human and chimpanzee [66]. The second proviral locus, Pan2Ap, was represented in the reference genome as a solo LTR and showed dimorphism between solo LTR and provirus, suggesting it is a recent germline acquisition. An interesting feature of the Pan2Ap provirus is that it possesses direct repeats of 15-18 bp, which are longer than the 4 to 6 bp target site duplications usually produced during retroviral integration [33]. All structural variants of the Pan2Ap locus retained the same direct repeats indicating their generation during proviral integration. Long direct repeat sequences have been observed to flank four human specific HERV-K(HML-2) loci and are likely to be a result of inefficient integration. They include three solo LTRs at: 7p21.2 (250 bp duplication); 6q21.1 (61 bp duplication); 17q22 (4 bp deletion) [4,60] and a Type I 3′ truncated provirus at 21q21.1 (450 bp duplication) [4]. Similar aberrant integrants have been observed *in vitro* in the Rous sarcoma virus (RSV)-derived RCAS vector system when the U5 region was mutated, thus serving as a poor substrate for viral integrase [67,68]. Subsequently host enzymes (DNA polymerases) were suggested to be involved in insertion [67].

The GAPS method is a refinement of RFLP and PCR suppression-based techniques developed to examine the diversity of HERV-K(HML-2) LTRs in hominoid genomes [42-46]. GAPS is different from these as it systematically avoids the amplification of SVA retrotransposons and HERV-K(HML-2) solo LTRs. Thus only full or near full-length HERV-K(HML-2) proviruses, containing *gag* or *env* genes, are amplified. Examination of the inter-individual genomic distribution of HERV-K(HML-2) proviruses has previously been addressed through southern-blot analysis [17] and high-resolution unblotting [23,40]. In these studies the distribution of HERV-K proviruses was consistent between individuals [17], with inter-individual differences attributed to solo LTR versus provirus allelism [23] and putative novel insertion events [40]. Here, we have combined the methodologies used in these previous studies with GAPS to isolate variable HERV-K(HML-2) proviruses in 145 humans, to our knowledge the largest representative set of genomes molecularly analysed to date. Our data supports the previously observed inter-individual distribution of HERV-K(HML-2) proviruses, but we observe that there are more variable proviral loci in humans than are represented in the human genome reference sequence.

Unlike studies utilising NGS to detect uncharacterised integrants, the targeted suppression PCR-based methodology applied in this article does not rely upon a reference sequence. Thus HERV-K(HML-2) loci present in unassembled regions such as centromeres, for example duplicated K111 sequences [69] and the provirus 19p12c characterised herein, can be detected. Furthermore polymorphism is detectable at all loci, which lie within the selective range of GAPS, not just at those absent from a reference genome. In our study, this is exemplified by the polymorphic ancient provirus 1p31.1a, which is represented by a solo LTR in the human reference genome. A further distinction of GAPS is the ability to target HERV-K(HML-2) sequence that are longer than 1kb. This allows proviral sequences to be selected for, and solo LTRs (~970 bp), which are provirus remnants, to be excluded. NGS-generated short reads are ideal for determining HERV-K(HML-2)-flanking sequence junctions [38,39,41,48] but independent locus specific PCR is still required to determine if integrants are solo LTRs or proviruses as most NGS system read lengths are too short to determine insertion structure.

GAPS isolates HERV-K(HML-2) proviral sequences from *Vsp* I restriction digests of genomic DNA. In estimating genome coverage we assume that we can detect HML-2 proviral insertions if they reside within ≤ 1 kb of a *Vsp* I site. Of the fifteen full-length human specific proviruses previously identified within assembled human genome sequences [4] we expected to isolate five, which are fixed or present at high frequency (K104, K106, K107, K108 and K109), from any given human sample. When applied *in vivo*, we detected four of these five proviruses suggesting an assay efficiency of 80%. Furthermore, on the basis that ten of the twenty six now known human specific proviruses were successfully recovered (25 described in [25,37] and one in this study), we suggest that with GAPS new HERV-K(HML-2) insertions can be detected from 38% of the human genome. Using additional, or more frequently cutting restriction enzymes could be used to increase genome coverage. However, GAPS will fail to detect proviral sequences that are distant from the chosen restriction site or contain deletions or mismatches in the primer sites.

GAPS is designed to select for proviral sequences, and as a consequence only inter-individual genetic variation for the presence or absence of proviruses is revealed. Subsequently, locus specific genotyping is required to determine if the observed variability is due to allelism for the pre-insertion site sequence or solo LTR formation. Notably, proviral loci that were haploid or diploid were equally amplified by GAPS (See Additional files 2 and 9 to compare the 3′ GAPS and 19p12c genotyping amplicons in the ECACC human ethnic diversity panel). This sensitivity suggests that the method could potentially detect proviruses present at lower than constitutive copy number, for example in DNA samples from individuals that are mosaic for HERV-K(HML-2) proviral insertions.

Within the present-day census of HERV-K(HML-2) proviruses in the human population none have the capacity to autonomously produce infectious particles [70]. Moreover, a HERV-K(HML-2) disease causing insertion has never been documented and the re-infection or retrotransposition of a naturally occurring provirus has never been observed *in vivo*. However, molecular reconstruction demonstrates that a recombinant could be weakly replication competent [15,16] and HERV-K(HML-2) viral RNA from the plasma of HIV-infected patients shows evidence of frequent recombination [14]. Analysis of polymorphism data suggests HERV-K(HML-2) mobility continuing until 250,000 years ago [41] and evolutionarily young HERV-K(HML-2) integrants are observed within archaic hominins [38,48] and in human populations [22-24,39,71], demonstrating recent proliferation. Using a cross-sectional dating method, three proviruses have been estimated to have entered the hominid germline at ~1.1 mya (K115) ~ 800,000 years ago (K113), and ~ 150,000 years ago (K106) respectively [65,72].

Determining if the HERV-K(HML-2) family is capable of re-infection or retrotransposition is of medical interest, as mobility could lead to disease through disruption of host genes or production of viral proteins. Such an infectious and replication competent HERV-K(HML-2) provirus is expected to be rare within the human population [24]. Moreover in order to corroborate the mobility of HERV-K(HML-2) it would be necessary to detect and subsequently independently verify the re-insertion of a viral copy within the same individual or diseased tissue. Such detection is technically challenging and would require single molecule sensitivity. Alternatively, assuming new integrations occur in germ cells and are passed onto subsequent generations as Mendelian traits, examination of pedigrees could be informative on the heritability and transmission of HERV-K(HML-2). In this study we applied GAPS to three CEPH pedigrees but did not observe any novel variation in the distribution pattern of the HERV-K(HML-2) complement (Additional file 2). This may simply be because an active HERV-K(HML-2) is likely to be rare and the families tested did not contain such a provirus. Second, the novel provirus may lie in a region of the genome not accessible to GAPS, due to restriction site distribution. Finally, the PCR primers used may not amplify a novel expanding lineage, but this seems unlikely as primers were designed to a consensus sequence and demonstrably amplify a wide age range of HERV-K(HML-2) proviruses.

Here we document a previously unreported ancient Type I HERV-K(HML-2) provirus that we named 1p31.1a, which occurs as a solo LTR or a provirus in humans. Based on the presence/absence of 1p31.1a at orthologous sites and estimated dates of speciation [73,74], 1p31.1a inserted and became fixed within the germline of the hominidae family before the diversification of the gorillini tribe 8.5-12 mya, but following the speciation of ponginae subfamily, 9-13 mya. Using LTR divergence as a measure of the relative provirus age we estimate that the chimpanzee ortholog inserted 7.87 (±2.78) mya. Due to LTR sequence homogenisation in recent human history, the human counterpart provided a much lower age estimate of 1.205 (±0.425) mya, showing that LTR divergence is not always a reliable indictor of proviral age. However, using LTR subgroup-specific variants, the human solo LTR of 1p31.1a (HML-2.LTR36) was calculated to have inserted 7.83 (±1.66) mya [37], congruent with our age estimate using LTR divergence in the chimpanzee provirus orthologue. Nevertheless, this age is slightly less than is expected based on molecular estimates of primate speciation dates. Comparative analysis of the Homininae subfamily 1p31.1a LTR sequences, presented here, indicate that they retain evidence of complex gene conversion/recombination events that may have altered the sequence features required to estimate time since insertion accurately. However, phylogenetic clustering of 1p31.1a LTRs shows that sequence homogenisation between proviral LTRs took place in the human lineage following diversification from chimpanzee. Based upon present day sequence similarity, this recombination event occurred ~ 6.5 mya after insertion of the provirus, between diverged LTR sequences. Intra-locus LTR gene conversion or recombination, leading to distortion of proviral LTR sequence, has been observed to have occurred at least three times in the HERV-K(HML-2) family; in the proviral loci K110 [58], 11q22 [23,61] and K115 [22]. Moreover a further five HERV-K(HML-2) proviruses have been the subject of ectopic recombination during primate evolution [75].

A more frequent consequence of homologous recombination between the LTRs of a provirus is deletion of internal sequence and replacement of a full-length provirus with a solo LTR sequence. In humans, variation in the form of solo LTR versus provirus allelism, has been observed in the endogenous retroviral families RTVL-H [76] and HERV-K(HML-2), where ten human specific loci vary in this way [4,23-25]. Solo LTR formation is predominantly a phenomenon of younger proviruses, whose LTR sequences have not diverged, and so it is hypothesised that the meiotic recombination process that generates solo LTRs requires a high degree of sequence similarity [61]. However, by contrast, an ancient HERV-H provirus that integrated prior to the diversification of gibbons, generated a solo LTR after the divergence of the human and chimpanzee lineages, many millions of years after its integration and fixation [77]. Similarly, here we describe an ancient HERV-K(HML-2) provirus (1p31.1a) that integrated and became fixed prior to the diversification of the gorillini tribe, which is variable for solo LTR versus provirus in humans. We detected the solo LTR in both African (3 individuals) and non-African individuals (2 Thai and 1 Australian aborigine), indicating that the solo LTR was generated prior to the emergence of anatomically modern humans from Africa. We propose that the sequence identity of the proviral LTRs rather than the local recombination rate, is likely to have enabled recombination-mediated deletion of proviral sequence leading to formation of a solo LTR. However, the low local recombination rate may have played a role in the persistence of the provirus in the full-length state for millions of years, prior to solo LTR formation.

Interestingly, analysis of HapMap data [78] shows that the region surrounding this ancient provirus (1p31) displays strong signals of very recent positive selection, specifically at *SLC44A5* within which another provirus, named 1p31.1b (also K116 and ERVK-1), is integrated. Further analysis of the 1p31.1a solo and proviral LTR haplotypes in multiple individuals could be informative, to identify recombination crossover points and determine if the solo LTR has formed more than once in recent human history, as has been observed for the human specific HERV-K(HML-2) provirus at 11q22 [23,61].

## Conclusions

In this study we applied a targeted suppression PCR-based methodology (GAPS) to examine inter-individual genetic variation of multiple HERV-K(HML-2) proviruses in humans and chimpanzees. Our results demonstrate that there are more polymorphic HERV-K(HML-2) proviral sequences within human and chimpanzee populations than are represented in genome assemblies. This result is not unexpected as reference genomes are demonstrably biased in favour of older or fixed retrotransposons and subsequently low allele frequency insertions are under-represented [39,52,79].

We observed a new polymorphic Type I HERV-K(HML-2) provirus, which is a recent addition to the human lineage, as evidenced by an intact pre-insertion sequence in humans and archaic hominins. However, this provirus is incapable of independent extracellular re-infection as it cannot encode a functional Env protein, due to a deletion at the *pol-env* boundary. We detected a species specific Type I HERV-K(HML-2) provirus in chimpanzee, showing that this subtype was also actively mobile within the germline of chimpanzees following, or around the time of, the Homo-Pan divergence. A further chimpanzee species specific HERV-K(HML-2) locus showed polymorphism for provirus versus solo LTR and possessed unusually long direct repeat sequences indicative of inefficient integration. We also characterised a previously unreported ancient HERV-K(HML-2) provirus which is variable for a provirus and solo LTR in humans. Such polymorphism is commonly associated with evolutionarily young or species specific integrants and has never been described at an ancient HERV-K(HML-2) locus. Moreover, we propose that evolutionarily recent sequence homogenisation of the ancient proviruses’s LTRs has facilitated the formation of a solo LTR within the human lineage.

Within the 145 humans examined here we did not detect any proviral insertions which were unique to an individual, suggesting that HERV-K(HML-2) proviruses do not significantly contribute to inter-individual genetic diversity, as has been observed for retrotransposon families such as L1, Alu and SVA [52,71,79-84]. Furthermore, if members of the HERV-K(HML-2) family are still actively mobile in the germline, our results indicate that such events are rare. However, we cannot exclude the possibility that we have failed to detect new insertions that reside in regions of the genome inaccessible to GAPS.

Further evidence of the mobility (or otherwise) of HERV-K(HML-2) sequences could be obtained by applying GAPS to cell lines or diseased tissues where retroviral particles have been observed, suggestive of activity in the soma. For example there is evidence of HERV expression in teratocarcinoma [7,85] and melanoma cell lines [9,86,87] and in the blood of HIV infected individuals [14]. Combining the targeted enrichment methodology of GAPS with analysis of the amplicons by NGS could be used to estimate the rate of re-insertion of HERV-K(HML-2), by comparing mobility within germ and somatic cells derived from the same individual. A similar approach has been applied successfully to detect somatic L1 insertion in human neurons [88,89].

## Availability of supporting data

The sequence of the 5′ and 3′ LTRs of proviruses 1p31.1a and Pan2Ap obtained in this study, have been deposited in GenBank under the accession numbers KP183324, KP183325, KP183326 and KP183327.

## Methods

### DNA samples and extraction

Lymphoblastoid cell line genomic DNAs derived from three CEPH/UTAH pedigrees (Families 1333, 1347 and 1424, a total of 44 individuals) was obtained from the Fondation Jean Dausset-CEPH. The ECACC Ethnic Diversity DNA panel, consisting of genomic DNA from 92 unrelated humans and a single common chimpanzee (*Pan troglodytes*) genomic DNA sample were purchased from the Health Protection Agency, UK. Buccal swabs from three unrelated common chimpanzees and one bonobo (*Pan paniscus*), were collected by the staff at Twycross Zoo using the Isohelix DNA Isolation Kit (Isohelix). Genomic DNA was extracted following the manufacturers protocol. Whole blood was collected from nine unrelated human volunteers of Northern European origin by venipuncture and genomic DNA isolated using the Gentra Puregene Blood kit (Qiagen), following the manufacturers protocol. These samples were combined to form a screening panel of 101 unrelated humans, 44 CEPH pedigree members and 4 unrelated chimpanzees.

### GAPS library construction and amplification

The GAPS method is a modification of the ATLAS technique [51]. Genomic DNA (300 ng) was digested to completion with 10-15 units of *Vsp I* (Promega) overnight at 37°C. Following digestion, the enzyme was inactivated by heating to 65°C for 20 min and 100 ng of digested DNA was ligated to an 80-fold molar excess of annealed linker. The linker was constructed by heating equal volumes of (50 μM) of the primers RBMSL2 and RBD3 (all primer sequences are listed in Additional file 11) at 65°C for 20 min and allowing them to cool to room temperature. Ligations were performed overnight at 15°C in 1 x Invitrogen Ligase Buffer (250 mM Tris-HCl (pH 7.6), 50 mM MgCl_2_, 5 mM ATP, 5 mM DTT, 25% (w/v) polyethylene glycol-8000) and 2-4 Weiss units of T4 ligase (Promega), followed by inactivation at 70°C for 10 min. Excess linker was removed using the QIAquick PCR Purification Kit (Qiagen), following the manufacturers protocol.

Suppression PCR was carried out using 2.5 – 5 ng of ligated genomic DNA, 1.25 μM of each primer, the first specific to the proviral region of choice (CMKGAG for *gag* and CMKENV for *env*) and the second specific to the linker (RBX4), in a final volume of 20 μl. Reactions also contained 1.1 x PCR buffer (45mM Tris HCl pH 8.8, 11mM NH_4_SO_4_, 5 mM MgCl_2_, 6.7mM β-mercaptoethanol, 113ug/ml BSA, 1.1mM dNTPs) and 0.5 units of *Taq* polymerase (ABgene). Amplification was performed in a MJ Tetrad PTC250, under the following conditions: 96°C for 1 min; 25 cycles of (96°C for 30 sec; 60°C for 2 min); and a final extension at 59°C for 10 min. The PCR products were diluted 1:50 in single molecule diluent (5 mM Tris-HCl (pH 7.5), 5 ng/μl sonicated *E.coli* genomic DNA) and a secondary PCR was performed under the same conditions using a primer specific to the HERV-K(HML-2) LTR (primer CMK5LTR for the proviral 5′ LTR and CMK3LTR for the proviral 3′ LTR) and a primer specific for the linker (RBY1). The secondary PCR products were size fractionated on 2% Seakem LE (Cambrex) 0.5X TBE agarose gels, visualised by ethidium bromide (0.5 μg/ml) staining and amplicons excised and purified using a QIAquick gel extraction kit (Qiagen), following the manufacturers protocol.

### Sequencing

DNA sequencing was carried out on purified PCR products using the Big Dye Terminator v3.1 ReadyReaction system (Applied Biosystems). Sequencing reactions were purified post-cycling using Performa DTR Filtration Cartridges (Edge BioSystems) and reactions were submitted to the Protein and Nucleic Acid Chemistry Laboratory (PNACL) at the University of Leicester, UK for data collection using an ABI3730XL capillary sequencer (Applied Biosystems).

### Sequence and phylogenetic analysis

The sequences of GAPS amplicons were visualised using the CHROMAS (Technelysium) sequence viewer and imported into the SIMMONIC sequence analysis package [90]. The chromosomal locations of proviral 5′ and 3′ LTR junction fragments were mapped to the draft sequence genomes of human (GRCh37/hg19; Feb. 2009), chimpanzee (CGSC 2.1.3/panTro3; Oct 2010), gorilla (gorGor3.1/gorGor3; May 2011) and Orangutan (WUGSC 2.0.2/ponAbe2; July 2007) using BLAT (http://genome.ucsc.edu) [91]. Proviral loci were verified using a unique junction sequence consisting of at least 20 bp of flanking sequence, a direct repeat, and 10 bp of the LTR. Orthologous sequence reads from the Denisovan and Neanderthal archaic human genomes were examined individually through selection of the full display of sequence tracks within CGSC 2.1/panTro2; Mar 2006 and GRCh37/hg19; Feb. 2009 [92]. Accession numbers were determined using the National Center for Biotechnology Information (NCBI) BLASTN program (http://blast.ncbi.nlm.nih.gov/Blast.cgi) [93]. Sequence co-ordinates of duplicated flanking sequences were examined in the database of genomic variation (http://projects.tcag.ca/variation/) [57].

A multiple alignment containing all LTR allelic variants of the proviral loci recovered using GAPS was generated using SIMMONIC [90]. An un-rooted neighbor-joining tree was generated from the alignment using MEGA 4 (http://www.megasoftware.net/) [94] with 500 bootstrap replicates, the Kimura two-parameter distance estimate [95] and pairwise deletion of gaps as parameters. Variable nucleotide substitutions within the homologous 1p31.1a LTRs were identified using the sequence data explorer in MEGA 4.

Relative proviral age (time passed since integration) was estimated by dividing the LTR divergence value by the nucleotide substitution rate of 3.77 x 10^−9^ ± 1.33 per site for each year [23] and calculating the mean. Pairwise distances between the 5′ and 3′ LTR of each provirus were calculated with the Kimura-2-parameter [95], using MEGA 4.

Gene density and local recombination rates were determined using the UCSC gene and Recombination Rate tracks within GRCh37/hg19; Feb. 2009. The default setting of the deCODE recombination rate [63] was selected.

### PCR genotyping

The proviral locus sequence, along with 2 kb of the flank, were submitted to Repeat Masker (http://www.repeatmasker.org/) and oligonucleotide primers designed to non-repetitive DNA using Primer 3 (http://biotools.umassmed.edu/bioapps/primer3_www.cgi). The primer sequences are listed in Additional file 11 and the combinations and annealing temperatures used for the amplification of each locus specific allelic variant are listed in the Additional file 12. PCR amplification was performed in 10 μl reactions containing 20 ng genomic DNA, 1.1 x PCR buffer (45mM Tris HCl pH 8.8, 11mM NH_4_SO_4_, 5 mM MgCl_2_, 6.7mM β-mercaptoethanol, 113ug/ml BSA, 0.5mM dNTPs), 0.625 μM of each primer and 0.02 Units of *Taq* polymerase (ABgene). Allele frequency was calculated in humans using the ECACC ethnic diversity panel (n = 92) and the panel of volunteers DNA samples (n = 9), for a total of 101 unrelated individuals. In chimpanzees presence or absence of a provirus was determined using a panel of four unrelated individuals. Gel extracted amplicons of the LTRs of proviruses 1p31.1a and Pan2Ap were verified by direct sequencing using the unique flanking and HERV-K(HML-2) primers CMKGAG, CMKENV, CMKF0001, CMK0520 and CMK3LTRAS.

## Additional files

Additional file 1:
**Agarose gels resolving GAPS amplicons from human blood and chimpanzee panel DNAs.** Un-cropped agarose gel images of those presented in main Figure 1. Lanes are as described in the Figure 1D legend in the main text. Additional file 2:
**Agarose gels resolving GAPS amplicons generated from the ECAAC human ethnic diversity panel and three CEPH families (1424, 1347 and 1333) DNAs.** (A) GAPS amplicons specific for the 5′ end of HERV-K(HML-2) proviruses. The top two gels in this figure show the amplicons generated from the ECAAC diversity panel (n = 92). There are four no DNA blanks within the ECAAC panel (F2, D6, H9 and H12) which have, as anticipated, not generated GAPS amplicons, these are denoted by a *. The lower gel picture shows the GAPS amplicons of three CEPH families (n = 44). The first lane of the control reactions contains a PCR reaction positive with the remaining lanes as described in the main text in Figure 1. The molecular weight marker was 100 bp ladder (NEB). (B) GAPS amplicons specific for the 3′ end of HERV-K(HML-2) proviruses. The top two gels in this figure show the amplicons generated from the ECAAC human ethnic diversity panel (n = 92). There are four no DNA blanks within the ECAAC panel (F2, D6, H9 and H12) which have, as anticipated, not generated GAPS amplicons, these are denoted by a *. The position of the 19p12c amplicon is highlighted on the left. The lower gel picture shows the GAPS amplicons of three CEPH families (n = 44) and one PCR reaction + ve. There are two ‘drop outs’, in family 1347 and 1424, that later produced the typical GAPS amplicon pattern upon repetition of the PCR reactions (data not shown). The control reactions are as described in the main text, in Figure 1. The molecular weight marker used was a 100 bp (NEB) ladder. Additional file 3:
**HERV-K(HML-2) proviral loci detected using GAPS.** ^a^Coordinates within hg19^1^, PanTro3^2^ , gorGor3^3^, or PonAbe2^4^. Additional file 4:
**Suggested nomenclature of HERV-K(HML-2) proviruses in chromosomal bands 1p31 and 19p12 in the human genome.** Nomenclature is consistent with [33] which is based upon cytogenetic positioning of a provirus within the human genome. Proviruses are annotated “a”, “b” etc depending upon their order within a cytogenetic band. Here, we suggest renaming provirus 19p12c (K52) to 19p12d, as we describe a new insertionally polymorphic provirus that lies upstream of 19p12c (K52) and downstream of 19p12b (K113). We further propose that provirus 1p31.1 (K4, K116, ERVK-1) be renamed 1p31.1b as we report an unassigned ancient provirus that lies upstream of provirus 1p31.1. Additional file 5:
**Alignment of HERV-K(HML-2) proviral sequences acquired using GAPS.** (A) Alignment of 5′ GAPS products. Alignment of GAPS sequences generated using a probe for the HERV-K(HML-2) *gag* region with enrichment for the 5′LTR. These GAPS products consist of the 5’ flank, direct repeat (pink) and start of the 5′LTR (yellow) of a selection of HERV-K(HML-2) proviruses which reside <1kb from a *Vsp*I (grey) restriction enzyme site. (B) Alignment of 3′ GAPS products. Alignment of GAPS sequences generated using a probe for the HERV-K(HML-2) *env* region with enrichment for the 3′ LTR. These GAPS products consist of the end of the 3′ LTR (yellow), direct repeat (pink) and 3′ flank of a selection of HERV-K(HML-2) proviruses which reside <1kb from a *Vsp*I (grey) restriction enzyme site. Additional file 6:
**Structure of orthologous 1p31.1a sequences.** (A) The human reference genome (hg19) contains a solo LTR and archaic hominin genomes (Vi33.16 and Denisova) retain the 1p31.1a locus. (B) The chimpanzee reference genome (PanTro2) contains a 1p31.1a provirus. Additional file 7:
**Agarose gel images of PCR genotyping for HERV-K(HML-2) proviruses in human blood and chimpanzee panel DNAs.** (A) 19p12c. (B) 1p31.1a. Un-cropped agarose gel images of those presented in main Figure 3. Lanes are as described in the Figure 3B legend, in the main text. Additional file 8:
**Agarose gel images of PCR genotyping for HERV-K(HML-2) proviruses in human blood and chimpanzee panel DNAs.** (A) Pan2Ap. (B) Pan8q. The locus specific primers A + B amplify the pre-insertion site and solo LTR. Primer combination A + GAG amplifies the 5′ LTR of a full-length provirus and ENV + B the 3′ LTR. Numbers on the left correspond to the co-migrating DNA size marker (kb). The identity of each band was confirmed by DNA sequencing. Additional file 9:
**Agarose gel images of PCR genotyping for dimorphic HERV-K(HML-2) proviruses in the ECAAC human ethnic diversity panel (Samples A1 to H12).** (A) 19p12c. The gel on the right shows amplification for the pre-insertion site using primer combination A + B. The left gel shows amplification with primers A + GAG for the provirus. There are four no DNA blanks within the ECAAC panel (F2, D6, H9 and H12) which have, as anticipated, not generated GAPS amplicons, these are denoted by a *. (B) 1p31.1a. The gel on the right shows amplification for the provirus using primer combination A + GAG. The left gel shows amplification for the Solo LTR using primers A + B. There are four no DNA blanks within the ECAAC human ethnic diversity panel (F2, D6, H9 and H12) which have, as anticipated, not generated GAPS amplicons, these are denoted by a *. Additional file 10:
**Genomic Location, gene density and local recombination rate surrounding the HERV-K(HML-2) proviruses 1p31.1a and 1p31.1b.** (A) UCSC Genome Browser screen capture of the ancient 1p31.1a locus. (B) UCSC Genome Browser screen capture of the human specific 1p31.1b locus. The human specific HERV-K (HML-2) 1p31.1b provirus is also variable for a solo LTR and a provirus and is located ~ 2Mb downstream of the ancient HERV-K (HML-2) 1p31.1a locus. The locations of the loci are highlighted in red circles. The figures show the genes and local recombination rates 1Mb upstream and 1Mb downstream of each of the loci. Additional file 11:
**Primers.**
Additional file 12:
**PCR Genotyping conditions.**

## Abbreviations

BLAT: BLAST-like alignment tool
CEPH: Centre d’Etude du Polymorphisme Humain
ERV: Endogenous retrovirus
Env: Envelope
Gag: Group specific antigen
GAPS: Genome-wide amplification of proviral sequences
HERV: Human endogenous retrovirus
HML: Human MMTV-like
LTR: Long terminal repeat
mya: Million years ago
NGS: Next generation sequencing
PCR: Polymerase chain reaction
RFLP: Restriction fragment length polymorphism
SVA: SINE-R VNTR-Alu
