# Supplementary material for: Genome-wide amplification of proviral sequences reveals new polymorphic HERV-K(HML-2) proviruses in humans and chimpanzees that are absent from genome assemblies
Source: Retrovirology. 2015 Apr 28;12:35. doi: 10.1186/s12977-015-0162-8 (PMC4422153; doi:10.1186/s12977-015-0162-8)

Additional File 1

Agarose gels resolving GAPS amplicons generated from human blood and chimpanzee panel DNAs

5′ GAPS

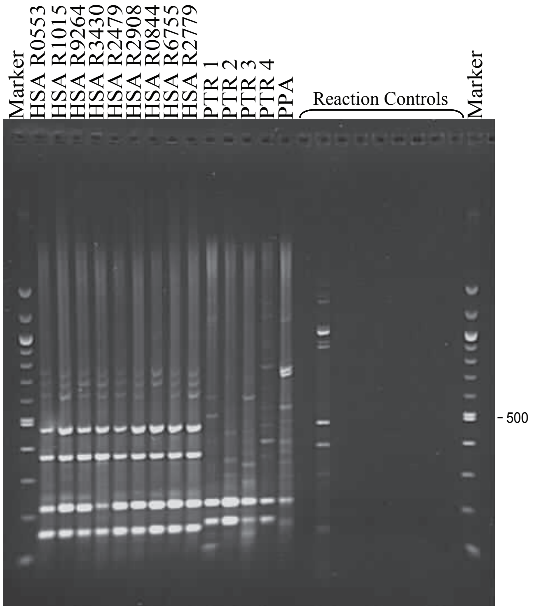

3′ GAPS

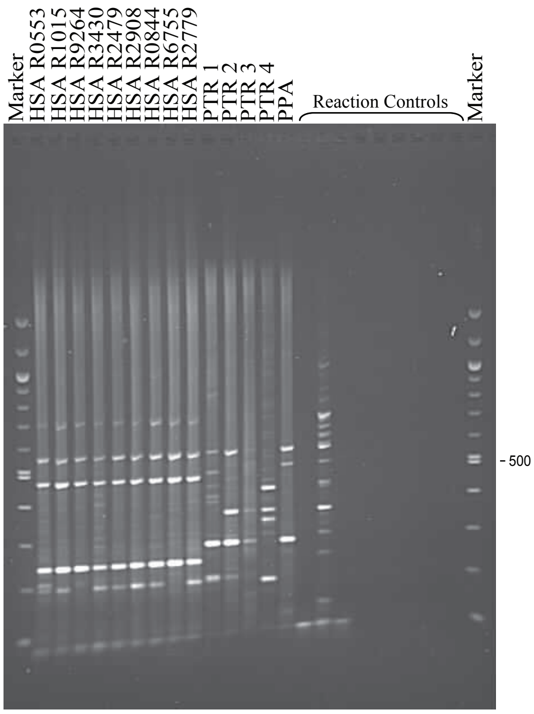

Supplement: Additional file 1: — Agarose gels resolving GAPS amplicons from human blood and chimpanzee panel DNAs. Un-cropped agarose gel images of those presented in main Figure 1. Lanes are as described in the Figure 1D legend in the main text. [file 12977_2015_162_MOESM1_ESM.pdf]
