# Supplementary material for: Genome-wide amplification of proviral sequences reveals new polymorphic HERV-K(HML-2) proviruses in humans and chimpanzees that are absent from genome assemblies
Source: Retrovirology. 2015 Apr 28;12:35. doi: 10.1186/s12977-015-0162-8 (PMC4422153; doi:10.1186/s12977-015-0162-8)

Additional File 2

Agarose gels resolving GAPS amplicons generated from the ECAAC human ethnic diversity panel and three CEPH families (1424, 1347 and 1333) DNAs

A

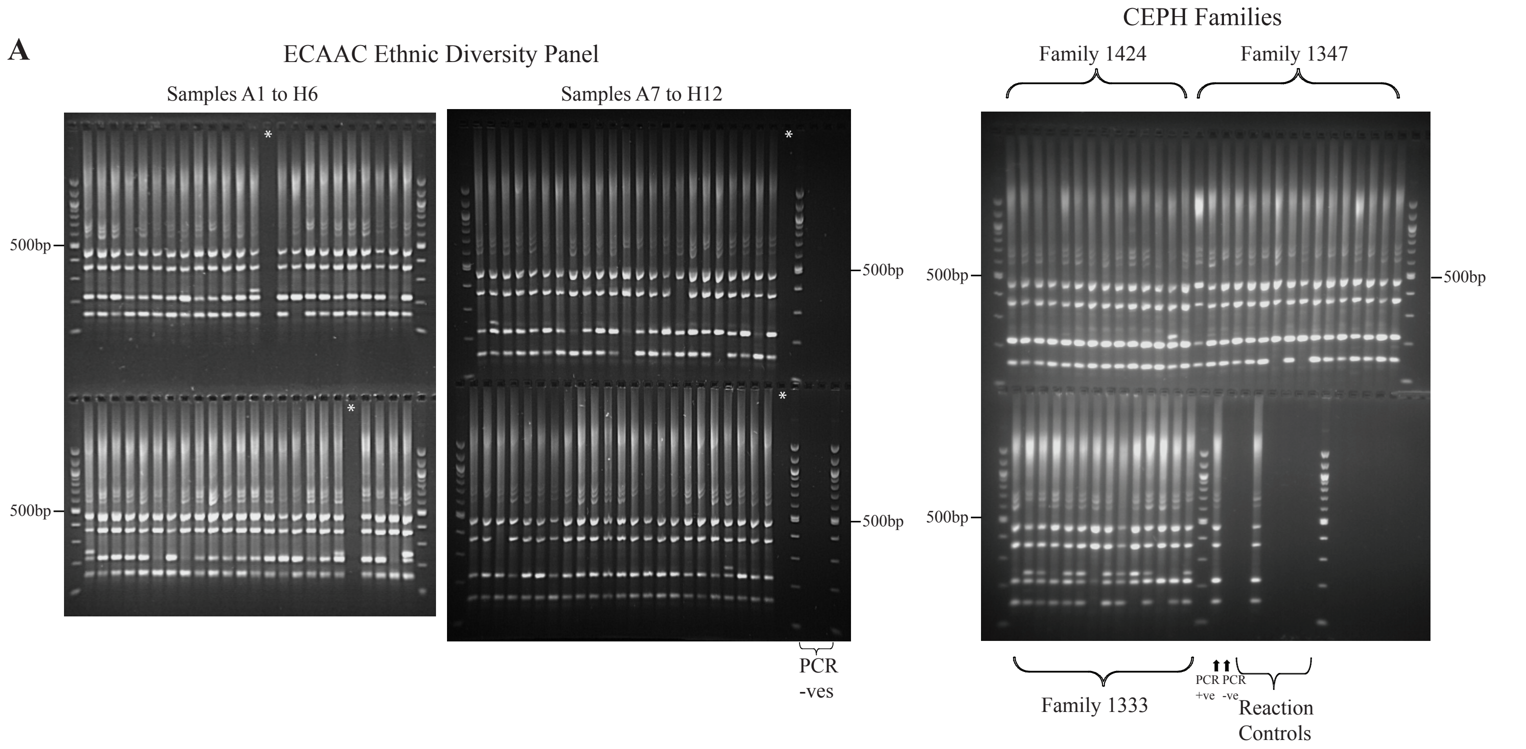

B

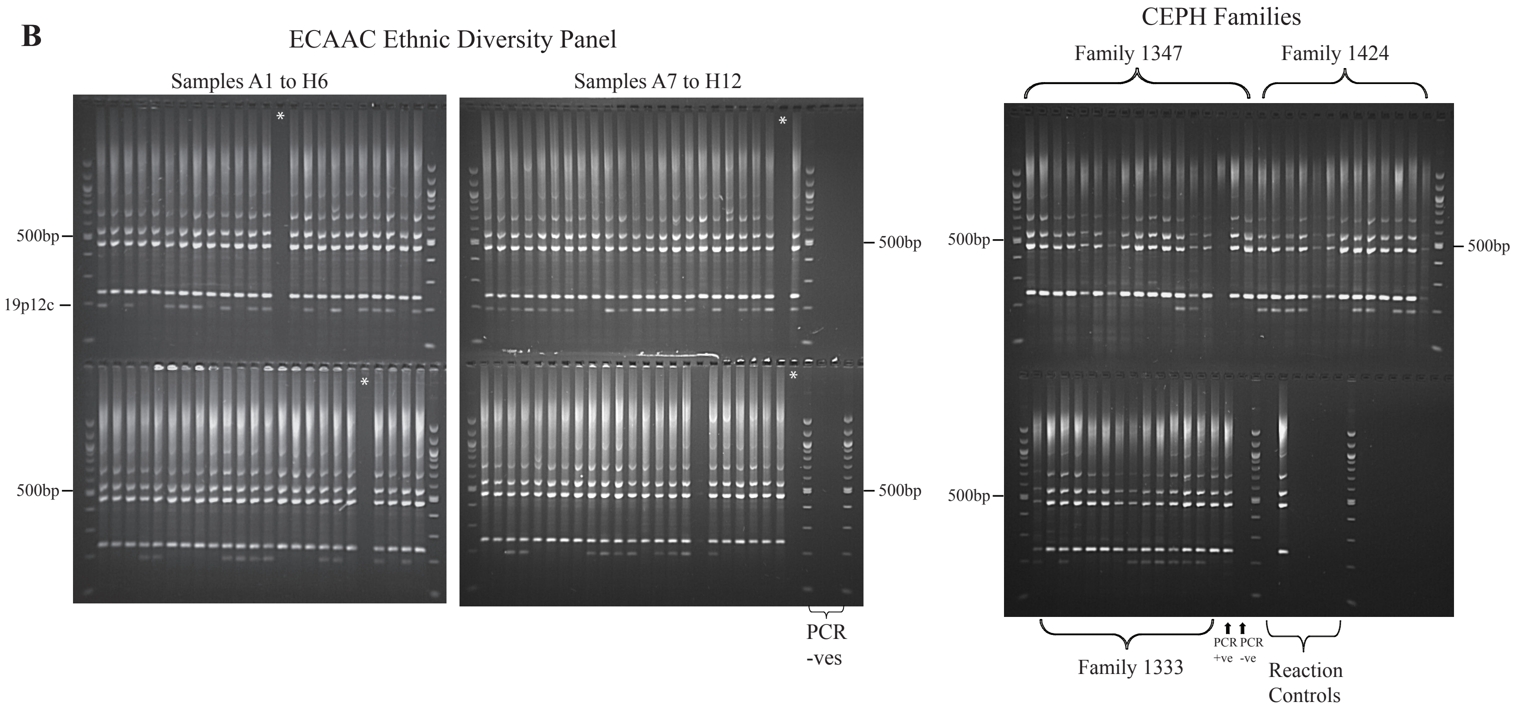

Supplement: Additional file 2: — Agarose gels resolving GAPS amplicons generated from the ECAAC human ethnic diversity panel and three CEPH families (1424, 1347 and 1333) DNAs. (A) GAPS amplicons specific for the 5′ end of HERV-K(HML-2) proviruses. The top two gels in this figure show the amplicons generated from the ECAAC diversity panel (n = 92). There are four no DNA blanks within the ECAAC panel (F2, D6, H9 and H12) which have, as anticipated, not generated GAPS amplicons, these are denoted by a *. The lower gel picture shows the GAPS amplicons of three CEPH families (n = 44). The first lane of the control reactions contains a PCR reaction positive with the remaining lanes as described in the main text in Figure 1. The molecular weight marker was 100 bp ladder (NEB). (B) GAPS amplicons specific for the 3′ end of HERV-K(HML-2) proviruses. The top two gels in this figure show the amplicons generated from the ECAAC human ethnic diversity panel (n = 92). There are four no DNA blanks within the ECAAC panel (F2, D6, H9 and H12) which have, as anticipated, not generated GAPS amplicons, these are denoted by a *. The position of the 19p12c amplicon is highlighted on the left. The lower gel picture shows the GAPS amplicons of three CEPH families (n = 44) and one PCR reaction + ve. There are two ‘drop outs’, in family 1347 and 1424, that later produced the typical GAPS amplicon pattern upon repetition of the PCR reactions (data not shown). The control reactions are as described in the main text, in Figure 1. The molecular weight marker used was a 100 bp (NEB) ladder. [file 12977_2015_162_MOESM2_ESM.pdf]
