# Supplementary material for: Genome-wide amplification of proviral sequences reveals new polymorphic HERV-K(HML-2) proviruses in humans and chimpanzees that are absent from genome assemblies
Source: Retrovirology. 2015 Apr 28;12:35. doi: 10.1186/s12977-015-0162-8 (PMC4422153; doi:10.1186/s12977-015-0162-8)
Supplement: Additional file 3: — HERV-K(HML-2) proviral loci detected using GAPS. aCoordinates within hg191, PanTro32 , gorGor33, or PonAbe24. [file 12977_2015_162_MOESM3_ESM.pdf]

Additional File 3

HERV-K(HML-2) proviral loci detected using GAPS

Loci	GAPS	Accession	UCSC Coordinates [a]	Features	References
K115	3′	AY037929	chr8: 7355397-7364859 [1]	Polymorphic for insertion	Turner et al., 2001
12q13	5′	AC122684	chr12: 55727215-55728183 [1]	Polymorphic for insertion, provirus and solo LTR	Belshaw et al., 2005
K104	5′ and 3′	AC025757	chr5: 30487114-30496205 [1]		Barbulescu et al., 1999
K106	5′	AC078785	chr3: 112743479-112752282[1]	Solo LTR versus provirus polymorphism	Barbulescu et al., 1999; Macfarlane and Simmonds, 2004
K107	5′ and 3′	AC016577	chr5: 156084717-156093896[1]	Solo LTR versus provirus polymorphism	Ono et al., 1986; Shin et al., 2013
K109	5′	AL590785	chr 6: 78427019-78436083 [1]	Solo LTR versus provirus polymorphism	Barbulescu et al., 1999; Shin et al., 2013
2q21.1	3′	AC018865	chr2:130719538-130722209 [1]	5'LTR and *gag* absent	Subramanian et al., 2011
7q22.2	5′	AC079796	chr7: 104388369-104393266[1]	3'LTR absent	Buzdin et al., 2007
4q32.1	5′	AC115222	chr4: 161579938-161582360[1]	3'LTR absent	Buzdin et al., 2007
19p12c	3′	AC008996	chr19: 22414379-22414383 [1]	5'LTR absent<br>Polymorphic for insertion in human	This Study<br>Contreras-Galindo et al., 2012
Pan8q	3′		chr8:112660047-112670551 [2]	*Gag* ORF disrupted<br>Chimp specific	This Study
Pan2Ap	3′		chr2a:9161883-9162878 [2]	Chimp specific<br>Solo LTR versus provirus polymorphism	This Study
4q32.3	5′x 2	AC106872	chr4:165916840-161582360 [1]	In human, chimp, gorilla and orangutan	Hughes and Coffin, 2001
K110	3′	AL121985	chr1:160660575-160669806 [1]	In human chimp and gorilla	Ono et al., 1986
1p31.1a	5′	AL606535	chr1:73594981-73595948 [1]<br>chr1:737777994-73787147 [2]<br>chr1: 75343534-75344061 [3]<br>chr1: 156,109,585 [4]	In human, chimp and gorilla. Solo LTR versus provirus polymorphism in humans	This Study
