# Supplementary material for: Genome-wide amplification of proviral sequences reveals new polymorphic HERV-K(HML-2) proviruses in humans and chimpanzees that are absent from genome assemblies
Source: Retrovirology. 2015 Apr 28;12:35. doi: 10.1186/s12977-015-0162-8 (PMC4422153; doi:10.1186/s12977-015-0162-8)
Supplement: Additional file 4: — Suggested nomenclature of HERV-K(HML-2) proviruses in chromosomal bands 1p31 and 19p12 in the human genome. Nomenclature is consistent with [33] which is based upon cytogenetic positioning of a provirus within the human genome. Proviruses are annotated “a”, “b” etc depending upon their order within a cytogenetic band. Here, we suggest renaming provirus 19p12c (K52) to 19p12d, as we describe a new insertionally polymorphic provirus that lies upstream of 19p12c (K52) and downstream of 19p12b (K113). We further propose that provirus 1p31.1 (K4, K116, ERVK-1) be renamed 1p31.1b as we report an unassigned ancient provirus that lies upstream of provirus 1p31.1. [file 12977_2015_162_MOESM4_ESM.pdf]

Additional File 4

Suggested nomenclature of HERV-K(HML-2) proviruses in chromosomal bands 1p31 and 19p12 in the human genome

Locus	Alias	Orientation	UCSC Coordinates (hg19)	References
1p31.1a		-	Chr1: 73594981-73595948	This Study
1p31.1b	1p31.1, K4, K116, ERVK-1	+	Chr1: 75842771-75849143	Hughes and Coffin, 2001
19p12a	K52	+	Chr19: 20387400-20397512	Hughes and Coffin, 2001
19p12b	K113	-	Chr19: 21841536-21841542	Turner et al., 2001
19p12c	19p12	-	Chr19: 22414379-22414383	This Study<br>Contreras-Galindo et al., 2012
19p12d	19p12c, K51	+	Chr19: 22757824-22764561	Hughes and Coffin, 2001
