# Supplementary material for: Genome-wide amplification of proviral sequences reveals new polymorphic HERV-K(HML-2) proviruses in humans and chimpanzees that are absent from genome assemblies
Source: Retrovirology. 2015 Apr 28;12:35. doi: 10.1186/s12977-015-0162-8 (PMC4422153; doi:10.1186/s12977-015-0162-8)
Supplement: Additional file 5: — Alignment of HERV-K(HML-2) proviral sequences acquired using GAPS. (A) Alignment of 5′ GAPS products. Alignment of GAPS sequences generated using a probe for the HERV-K(HML-2) gag region with enrichment for the 5′LTR. These GAPS products consist of the 5’ flank, direct repeat (pink) and start of the 5′LTR (yellow) of a selection of HERV-K(HML-2) proviruses which reside <1kb from a VspI (grey) restriction enzyme site. (B) Alignment of 3′ GAPS products. Alignment of GAPS sequences generated using a probe for the HERV-K(HML-2) env region with enrichment for the 3′ LTR. These GAPS products consist of the end of the 3′ LTR (yellow), direct repeat (pink) and 3′ flank of a selection of HERV-K(HML-2) proviruses which reside <1kb from a VspI (grey) restriction enzyme site. [file 12977_2015_162_MOESM5_ESM.pdf]

Additional File 5
Alignment of HERV-K(HML-2) proviral sequences acquired using GAPS

A

```
            1                                                                                                                    120
            |                                                                                                                      |
4q321       ATTAATGGTGAGTGTGGAATAATTCACTTATCTTGTGTATTTGATGATTATAACAAGTCAGACTCTACATACTTTACATTTTTAAGAAAATAGTGCTAAAGTTACTTTTAGAATGAGTGG

            121                                                                                                                  240
            |                                                                                                                      |
4q321       GACTTTCCAACTCACATATTCAGTCATAGCAGGTAACACTTACACAGAGATACAGATTATAAGACGAGTATATAAATGGCTGTTTCAATTCTAACAAAAGTACCAGGAGTTATATAGTAG

            241                                                                                                                  360
            |                                                                                                                      |
4q321       TGTCTCTAGTGTTTACAATACTCAGGCAATTGAAAGAAAAAAAGACTTTGTTTAGTCTAAACTAAAATGTGTTTTGAGAATACATAACTACTTGTAAGTAGGCTTGGATATCTTTTCAGA
4q323long                                      ATTAATCTAGAAGACTGGATACCCTACAGATTGTTCAGAACACATCTATTTCATTTATAGTATTTCCCAGAGAGAATCTTTCTCA
K107                                                                                                            ATTAATCTTATAAGTG

            361                                                                                                                  480
            |                                                                                                                      |
4q321       AAAAGTTAAGACAGATGTAAATAATATGAAAAGAGAAATGCATATAGATTTTTTAGATGAAAGAGGGGAGCACACAGCATCCCAAATTGTGATATCGTTTTTGCCTAAGCACCAGGGGTT
4q323long   AATTGGGTAATCCATTAAAAAATAGCCCGAAGGCTGGGCGCGGTGGCTCACACTTGTAATACCAGCACTTTGACAGGCCGAGGTAGGCGGATCACCTGAGGTCAGGAGTTCAAGACCAGC
K107        CAAAACAAAGCTAAAAACTTTAAGTACATGGATTGCTGGGGTGTGTGGGGAAGTGGAGAGAAGTAGAGAGGAAGACAATTACAGAACATGTTTGCAAACTGTTCAAAAGGGAGAAAATTT

             481                                                                                                                  600
            |                                                                                                                      |
4q321       TTAGGGAAAAAGACACGTTCAGTGAATAACACCGTGAAATAAATTCATTGAATGTTTGTTTTTAAATTTTTAAAATAAATCCTGAAAGTTTCTAGTGAAGTATACAAAGAATATTGTCTA
4q323long   CTGGCCAACATGGTGAAACTTCGTCTCTACTAAAAATACAAAAATTGGCCTGGCCTGGTGGGCACCTGTAATCTCAGCTACTGGAGTGGCTGAGGTAGGAGAATTGCTTGAACCCAGGAG
K107        ACGGATTTCTTCCACAATGGTATCAGTCATACTGCCCCACATACCAAGTACCTACAGCTAAGAAGCTCAATATACACTCCTCCTTGTGTCTGTCTGTCTCTCTGTCTCTTTGTATGTGTC
1p311a                                                                   TAATTAGGAAAGAAGGTAAAGGAGAG.ATGAAGAGAGTAAAAAGAAGAAAACAAACAAA
K104                                                                                                       ATTAATAGCCTACCAACTAACCAAAGTCC

            601                                                                                                                  720
            |                                                                                                                      |
4q321       TTCTAAGGCAGCAGAGAGAGCATGCCTGGAACAGAAATTGTCTGCTGACTACGGGGTTCAAGGTTTCTTACTCAACTGTATGGAAGAAATAATCTGACTGGAGAGAAGACTGAGATGAGG
4q323long   GAAGAGGTTGCAGTGAGCCGAGATTGCGCCACTGCACTCCAGCCTGGGTGACAGAGCAAGATTCCGTCTAAAAAAAAAAAAAAAAAAAAAAAAAAAGAGCCTGAAGTATATATAGTGATAT
K107        TTTCTCACTTTCAATATATATGTTTATACAGAATGAGGATCCTTTATCTGAAATAAGGGACCAGAAGTGTTTTGAAATATGAATTTTTCCAGATTTTGAAATACCTGCATTACAGGTTGA
1p311a      TTAAAAGTACCTACTTTAAAGAGTACTTTAGCAATTCAATCAAGTTTTTGATACATTCTTACAATAACCGTAGGAAGCAGAATTATTATGATGTTTTACTACAGTTGAAAAATTAGATTT
K104        AGGACTAGATGGATTCACAGCCAAATTCTACCAGAGGTACAAAGAGGAGCTGGTACCATTCCTTCTGAAACTATTCCAATCAATAGAAAAAGAGGTTATCCTCCCTAACTCATTTAGTGA
K106                                                                                  ATTAATGTCTACAGCCAGTTGATTTTATGTAAAGGGATTATT
```

```
               721                                                                                                                  840
               |                                                                                                                      |
4q321          AAAGAAATTTCTGAATCAGTACCATGCCTTTTGTTTGTTTGTTTATTTATTTGTTTTTCTTCCTTCTCAGAATTACTAAAAGAATAGAATACCAAAAAGGGAACAATGATGGCCGAGTTT
4q323long      AGGACAAAAATAAATGATAAGTAGGCAGTTGCAGCATATGCAAGTAGCTCCCATTTGGAGATAAACAAAGGGGACCATTCTATACATAACCTATATTCATATAAATGTTTTATTTTTGTA
K107           GCGCCCCAAATCTGAAAATCTGAGATTTGAAATGCTCCAATGAGTATTTCCTTTGAGCTTTATGTCAGTCCTCAAAAAGTTTTCAATTTTAGAGCATTTTGGATTTTGGATTTTTGGATT
1p311a         AGAGAGGCTAAATGTTTTACTTTTCTCCTAGTGACATAAATTATGAACTGTAGAAAAGAGCCCAACTCTTAGATTTTTCTCCCCAAACACACGGGCTGGTCCACTTTACCCAATGGTAGA
K104           AGCCAGCATCATCCGGACACCAAAGCCCGGCAGAGACACAACAAAAAAAAGAGAATTTTAGACAAATATCCCTGATGAACATCAATGTGAAAATCCTCAATAAAATACTGGCAAACAGAA
K106           CTGGGTAATGTGGGTGAGCCTGATCCAATCAGTTGAAAGGCCTAAAGAATAGAACTTAGGTTTTCCTGAAGATGTGAAGAAAGAAGAAATTCCACCTGCGGACTTCCTCTTCAGCTCCTG
7q222                  ATTAATCCCTTCCTTCACCAGCTAATGCACACCCACTAAGTGACACCCACAGCAAGCCTTATCATTATTATTATGCAAATAATAATTTTCAGGTAGTAGCTATGGAGCTTCT
12q13                                                                                                                      ATTAATGGCTGG

               841                                                                                                                  960
               |                                                                                                                      |
4q321          CCTCTTAGAGGACAGTACTGATCCATCTGTGTCTTTGCGTATGGTTGTGAAGGTGATACAGGCCAGGTTTACCATCCATCTGACCCTTCATCATAACAAACAAACCTATGTTCTTTGTGG
4q323long      TATGCATAGACCATTTGGGAATACTTACAAAAAGCCATTAACAGCAATTATCCTTTAGAAGGAAAATGAGTGACAGCAAGTTACTTTGTAACTGTTGTGCCAGGTAGGGCTTTCT.....
K107           TGGGACACCCAACCTGCATGGTTTTTAATTTTAATTTTTATTTATTATTAGTTTTTGAGACAAAGTCACTCTGTCACCCAGGCTGGGGTGCAGTGGCATGATCTCAACTCACTG.....
1p311a         TCACAAAATCCCATGATGGGTAACAGTGAAGAACCAGTGACTGCCTCAGGTTTCCATTTGGGGTCCTCATGAAGCAGAATGTCTCTTCATCTTTTGGGAGATCAACAACTCATGT.....
K104           TCCAGCAGCATATCAGAAAGGTTATCCACCACGATCAAGTCAGCTTCATCCCTGGGATGCAAGTCTTGTTTAACATACACAAATCAATAAACATAATCCATCATATAAACAGAAC.....
K106           CCTGAGAGTTTCCAGCCTTATGAATTTCAGACTTTCCTATCTGACCTCTACAATGGTATAAGTCAATTTCTTTCTGTAAATCCCTTTACATATTCTACTGTTTCTCTTTGGCTGG.....
7q222          AAGAGCATAATATTTACTTGGGATACTTATCTTAAATGCAAATTCTGACTTCAGTAGTTGTAAGGAACAGCTCTGTAATCTTTTTTTCTTCAATTAATAAATTGTATTTTTAGAG.....
12q13          ACTATCGGAGAATTCCACCTTCAAATGTTAATTTTACTCTCATGTTCTCCCGAATGATGTTGATAAAAAAACTTTAGGTCTAGCACACTTTATCTGTAAATTTGTATACTGCTAT.....
4q323short                                         ATTAATAGCAATTATCCTTTAGAAGGAAAATGAGTGACAGCAAGTAACTGTGTAACTGTTGTGCCAGGTAGGGCTTTCT.A...
K109                                                                 ATTAATATTCTATGACACTCACTGCCAGTCTCAGGTGTTTGGATCTTCCACATATATATGC.....

               961                                                             1028
               |                                                                  |
4q321          GGAAAAGCAAGAGAGATCAGATTGTTACCGTGTCTGTGTAGAAAGAAGTAGACATAGGAGACTCCATT
4q323long      .......A......--............T.......................................
K107           ...................A........T.......................................
1p311a         .......A....................TA......................................
K104           ............................T.......................................
K106           ............................T.......................................
7q222          ............................T.......................................
12q13          ............................T.......................................
4q323short     .......A......--............T.......................................
K109           ............................T.......................................
```

B

```
          1                                                                                                                      120
          |                                                                                                                        |
Pan8q     GATCCTCCATATGCTGAACGCTGGTTCCCCGGGTCCCCTTATTTCTTTCTCTATACTTTGTCTCTGTGTCTTTTTCTTTCCTAAGTCTCTCGTTCCACCTTACGAGAAACACCCACAGGT
Pan2Ap    ........G...............................................................................................................
K107      ...............................................................................T.C..A........C..........................
K110      ....................T............C.............................................T.C....................T.................
2q211     .........................C...T................................................................A.........................
K104      ................................A..........................................................A............................
K115      ..................T............................................................T.C..A........C..........................
19p12c    ..................T.......T.....T..............................................T.C..A........C.........A................

          121                                                                                                                    240
          |                                                                                                                        |
Pan8q     GTGGAGGGGCAACCCACCCCTACAAGGAGATACCATCTCACACCAGTTAGAATGGCAATCATTAAAGTCAGGAAACAACAGGTGCTGGAGAGGATGTGGAGAAATAGGAACACTTTTACA
Pan2Ap    ...T....................GCTGATGGGCGAGAACTGTGAGAATCTCTTCATTCAGGCATGCACAGCCCACAAGTGTGGGGGCTTAACACTCCACTAGACCAGTGGGGTGCAGGA
K107      ...T....................ACTGCAACCTCCGCCTCCTAGTTCAAGTGATTCTCCTGCCTCAGCCTCCCAACTAGCTGGGATTACCGGCATGCACCACCACACCCAGCTAATTTT
K110      .....................T..TGAGACAATATTTAAAGGTTTGGGGAAATCCTGTAAGGCAGTAATCACAGCAATTAACTCCGCCTTTTGAGCAGAAGTATAAGAGGTAGAAATAAG
2q211     ............T........T..AGAACTTAATAGTAGGCCCAATTGATAAACTAACAGCGTTTCCTATATTCAACATCTTTATGATTCCATGCAGTCTAAGCATGGTTTAAAGCCACG
K104      ........................CAGAACCAATGACAAAAACCACATGATTATCTCAATAGATGCAGAAAAGGCCTTCGACAAAATTCAATAGCCATTAAT
K115      .G.T....................CCTTTTATATTGATCATTAAATTCACTTCTAACTCTGAGATTCTATGTGATTAAAT
19p12c    ...T...............T....TCTTTGACATCTTCTGCTCTAATTTAATTAAT

          241                                                                                                                    360
          |                                                                                                                        |
Pan8q     CTGTTGGTGGGACTGTAAACTAGTTCAACCATTGTGGAAGTCAGTGTGGCAATTCCTCAGGGATCTAGAACTAGAAATACCATTTGACCCAGCCATCCCATTACTGGGTATATACCCAAA
Pan2Ap    GCCAATGAATACATGCTTCTCCCTTTGAACCCTGTGCAAAGTCCTGAGATGCATTTCCTCAAAGATCTCATGATCCTCACATGTCATGATCCCATAAGACTGGGAGCTGGTTGCCTCTAG
K107      TGTATTTTTAGTAGAGATGGGGTTTCACCATGGTGGCCAGGCTGATCTGGAACTCCTGAGCTCAGGTGATCCTCCTGCCTCAGCCTCCCAAAGTGCTGGGATCATAGGCATGAGCTACCC
K110      TTTGTCTGTAGGACTTACATAGCCAGCATTGCCATTACTGGAGCCATCAGTGAACACTGTAACGGCCTCAGGAATGGGTTGATTTTTGGTTAATCGAGGAACCACCTAAGAAGTCATTTT
2q211     GTGTTAACATAAGCCTGAGTTAGGTTTAAGAGGTTATATTTCAGATGTGTAAAGAGGCTTTTTAGCAAAACAACTTGCAATCTCAGACAGCCCTTATATCTTCACACTGAGGGTCTGAGA

          361                                                                                                                    480
          |                                                                                                                        |
Pan8q     GGACTATAAATCATGCTGCTATAAAGACACATGCACACGCATGTTTATTGCAGCACTATTCACAATAGCTAAGACTTGGAACCAACCCAAATGTCCAACAATGATAGACTGGATTAAGAA
Pan2Ap    TGGTAGCTGTTGGGGAAATATAATTAAAATCAAAATCTCCCAAACCAGAAATCTTCTCTACAGTGGTAATATAGGAAGAAAACACCTTTACTAATGAATAAGAAACAAACTAGAATATCA
K107      CACACCTGGCCTCAACCTGTATGTTTTTAATGAGAACACATGGACACAGGAAGGGGAACATCAC.CTC.GGGGACTACTGTGGGGTGGGGGGAGGGGGGAGGGATAGCATTAGGAGATAT
K110      TATAAAATCAAATAATTTGTTTTTTGGATAATGATTGTCAATAACGCCAATAAAATCAGCCAAGTGAATTTGCCACAGTACAGAATGTTGAAAGGCAGCTTGAACTTTGAGCCAATTTAA
2q211     ACATGAAAGAGAAGTCAAAGAACCTGCAACTGAGGTCAGAGAGCACAGTGCAATCACATTTTTATCTTGCTAGGGCCCTTTCTGGAGTCTCATTAAT

          481                                                                                                                    600
          |                                                                                                                        |
Pan8q     AATGTGGCACATATACACCATACTATGCAGCCATAAAATACTATGCAGCCATAAAAAATGATGAGTTCATGTCCTTTGTAGGGACATGGATGAAACTGGAAATCATCATTCTCAGTAAACA
Pan2Ap    TGCACATCACACGGCCATCATACAGGATTGCAAAGGCAGAAATAATGCTCACCTCTTTATGTACCCAAGCATATACGACCCATCACATACATGTTCTCAAGATAAACAATAAATCATCCTC
K107      ACCTAATGCTAAATGACCAGTTAATGGGTGCAGCACACCAACATGGCACATGTATACAATATGTAACAAACCTGCACATTGTGCACATGTACCCTAAAACTTAATGTATAATAATAATAAA
K110      AGGAACTACAATTATATTTGGATCAAATCCAGAAATTTGAAGTATTCTGCACCAAGCCATTCTAATTAAT
```

```
         601                                                                                                                      720
         |                                                                                                                        |
Pan8q    TATCGCAAGGTCAAAAAACCAAACACCGCATGTTCTCACTCATAGATGGGAATTGAACAATGAGAATACATGGACACAGGAAGGGGAACATCACACTCTGGGGACTGTTGTGGGGTGGGG
Pan2Ap   AAGTAAAAGGACTTGACAGCACCATTTGCCACATGTAGTTCATCCTCTTAAATTCATCTGGTAATTGTGGTAACCATCTAGGTTAGCTTATTAGCTTTATCCAGTGGAAAAATTAAT
K107     ATAAAATAAAAGAGAGAGGAAAAAAAAAAAAGTCAGACTAGTTATCCAATATTTTGGTACTGTAACTGTCAGTGGTGTGCGGAGAATTACCAAGCCTCTAATTAAT

         721                                                                                                                      840
         |                                                                                                                        |
Pan8q    GGAAGGGGGAGGGATAGCATTAGGAGATATACCTAATGCTAAATGACGAGTTAATGGGTGCAGCACACCAGCATGGCACATGTATACATATGTAACTAACCTGCACATTGTGCACATGTA

         841                                                                                                                      960
         |                                                                                                                        |
Pan8q    CCCTAAAACTTAAAGTATAATAATAATAAAAAAATTTGAAAGAAGTGACTTACACCAGATGCACAGAAATCAAGGTAAGGGCACAAAAAACATTATAAAAAAATATATATAAAAACAAGG

         961                                                                                                                      1080
         |                                                                                                                        |
Pan8q    AAACATGAGACTTCCAAAGGAACAAAATAATTCTTCATCAGTAAATTCCAATAAAAAATGTATAAACTGCCTAAAAGATAATTGAAAACAATGATATTGAATAAGCTCAGTGAGACACAA

         1081                                        1125
         |                                           |
Pan8q    GAGAACATAGATAAACAGTATGAAGAAATCAGAAAAACAATTAAT
```
