# Supplementary figures and images for: Genome-wide amplification of proviral sequences reveals new polymorphic HERV-K(HML-2) proviruses in humans and chimpanzees that are absent from genome assemblies
Source: Retrovirology. 2015 Apr 28;12:35. doi: 10.1186/s12977-015-0162-8 (PMC4422153; doi:10.1186/s12977-015-0162-8)

Additional File 6

Structure of orthologous 1p31.1a sequences

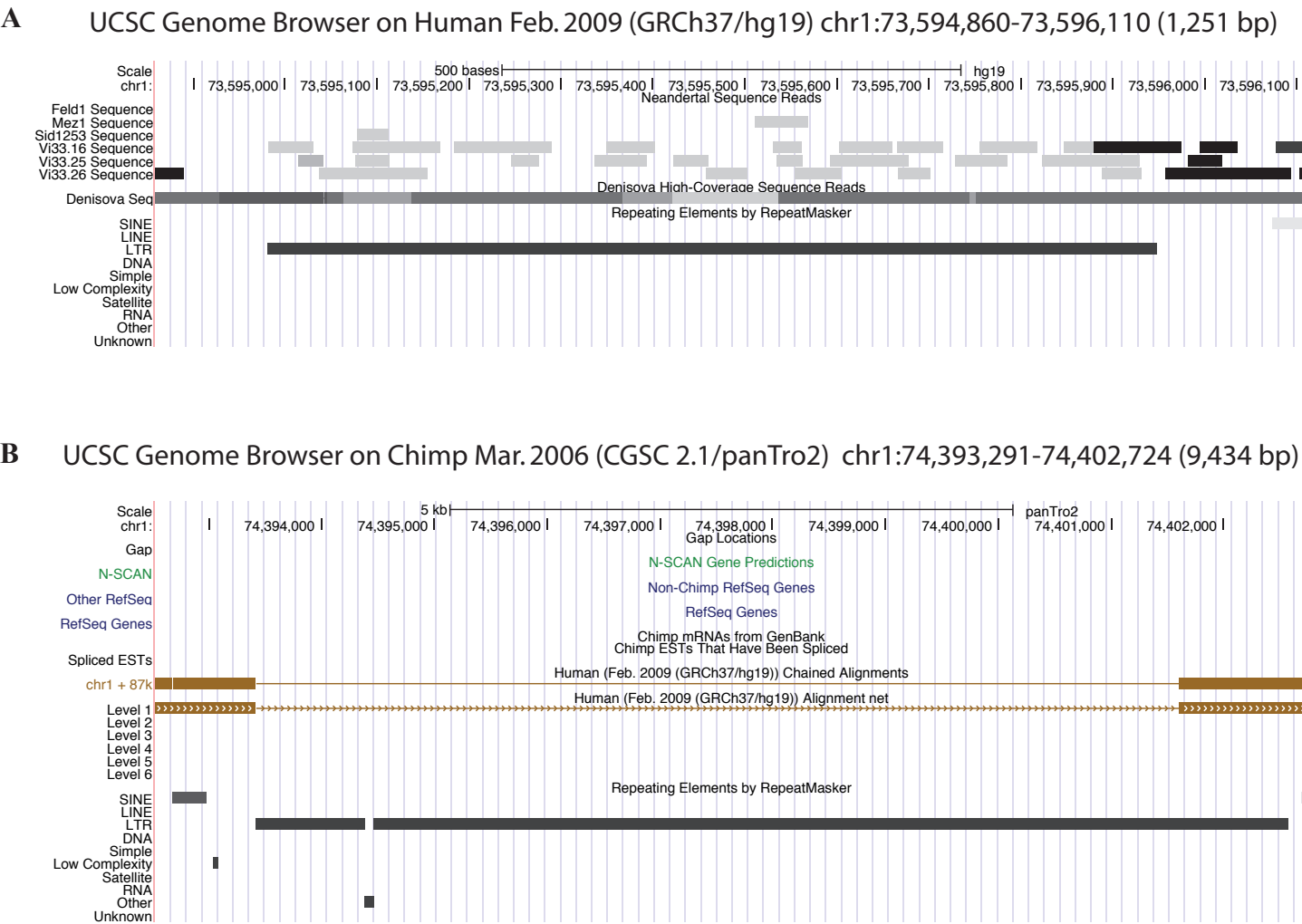

Supplement: Additional file 6: — Structure of orthologous 1p31.1a sequences. (A) The human reference genome (hg19) contains a solo LTR and archaic hominin genomes (Vi33.16 and Denisova) retain the 1p31.1a locus. (B) The chimpanzee reference genome (PanTro2) contains a 1p31.1a provirus. [file 12977_2015_162_MOESM6_ESM.pdf]
