# Supplementary material for: Genome-wide amplification of proviral sequences reveals new polymorphic HERV-K(HML-2) proviruses in humans and chimpanzees that are absent from genome assemblies
Source: Retrovirology. 2015 Apr 28;12:35. doi: 10.1186/s12977-015-0162-8 (PMC4422153; doi:10.1186/s12977-015-0162-8)

Additional File 7

Agarose gel images of PCR genotyping for HERV-K(HML-2) proviruses in human blood and chimpanzee panel DNAs

A 19p12c

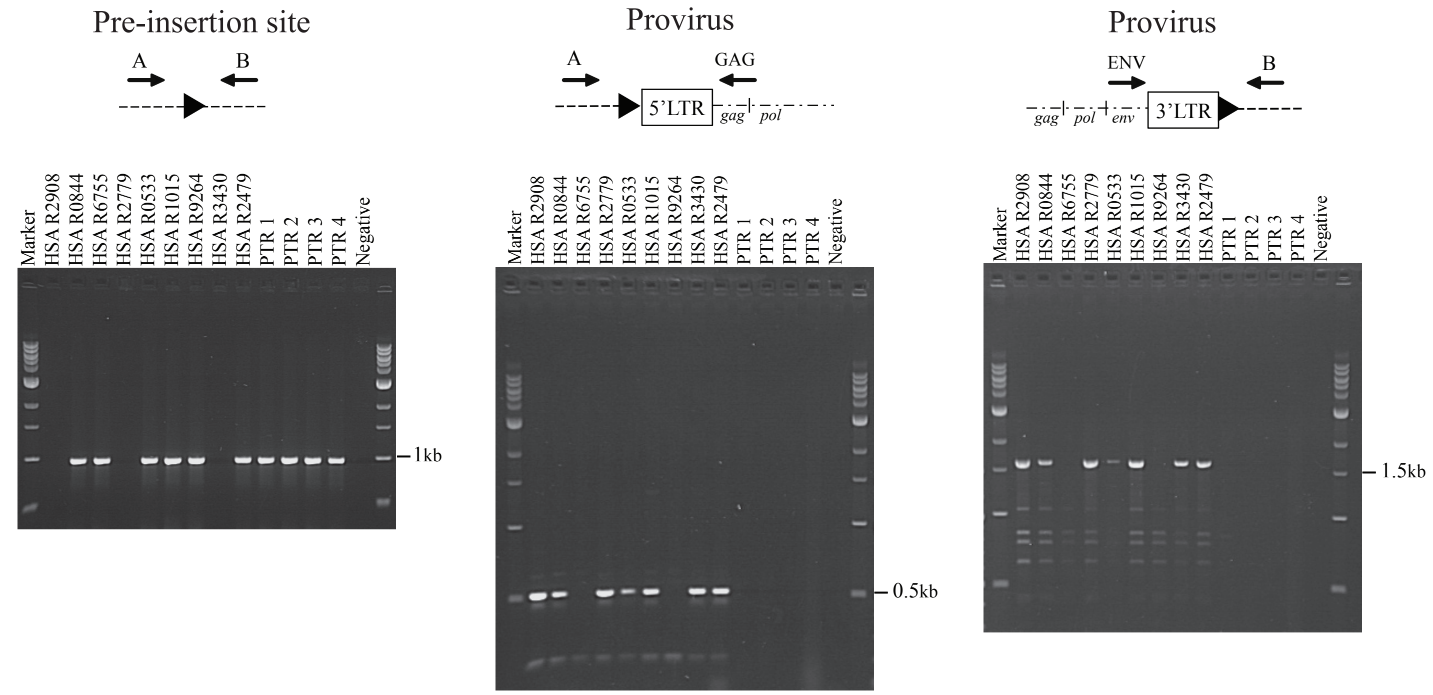

B 1p31.1a

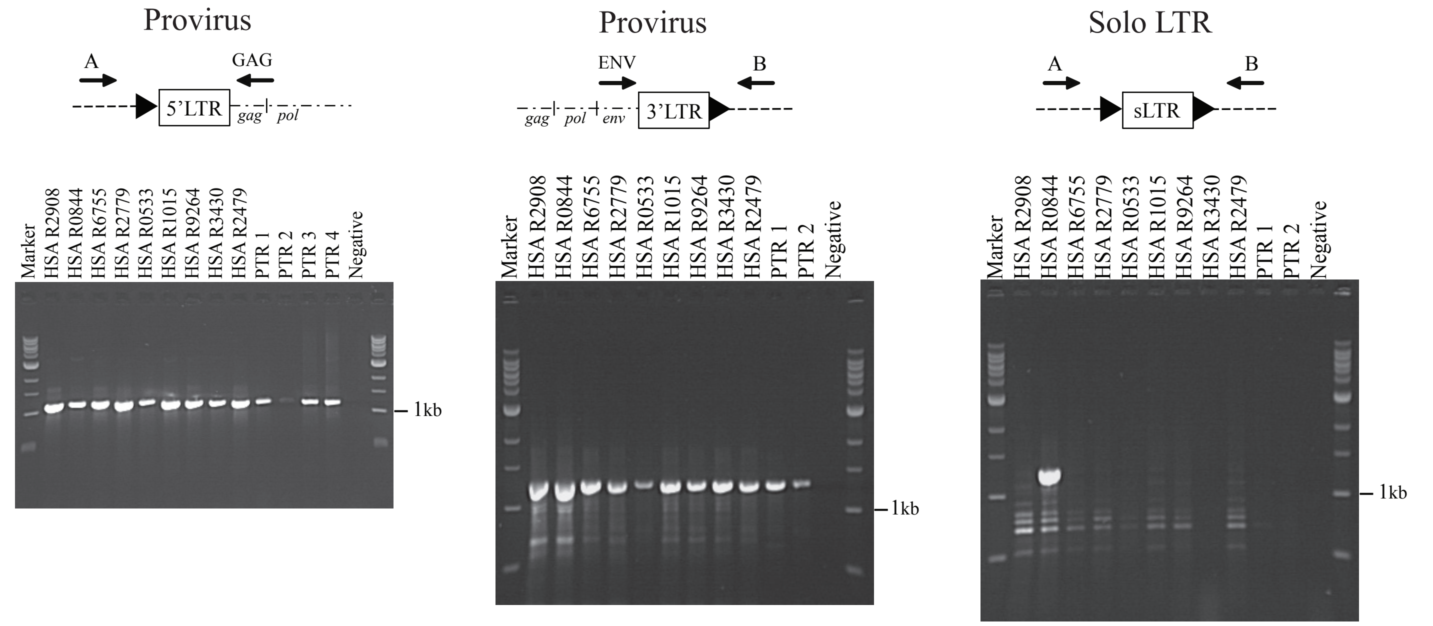

Supplement: Additional file 7: — Agarose gel images of PCR genotyping for HERV-K(HML-2) proviruses in human blood and chimpanzee panel DNAs. (A) 19p12c. (B) 1p31.1a. Un-cropped agarose gel images of those presented in main Figure 3. Lanes are as described in the Figure 3B legend, in the main text. [file 12977_2015_162_MOESM7_ESM.pdf]
