# Supplementary material for: Genome-wide amplification of proviral sequences reveals new polymorphic HERV-K(HML-2) proviruses in humans and chimpanzees that are absent from genome assemblies
Source: Retrovirology. 2015 Apr 28;12:35. doi: 10.1186/s12977-015-0162-8 (PMC4422153; doi:10.1186/s12977-015-0162-8)

Additional File 8

Agarose gel images of PCR genotyping for HERV-K(HML-2) proviruses in human blood and chimpanzee panel DNAs

A Pan2Ap

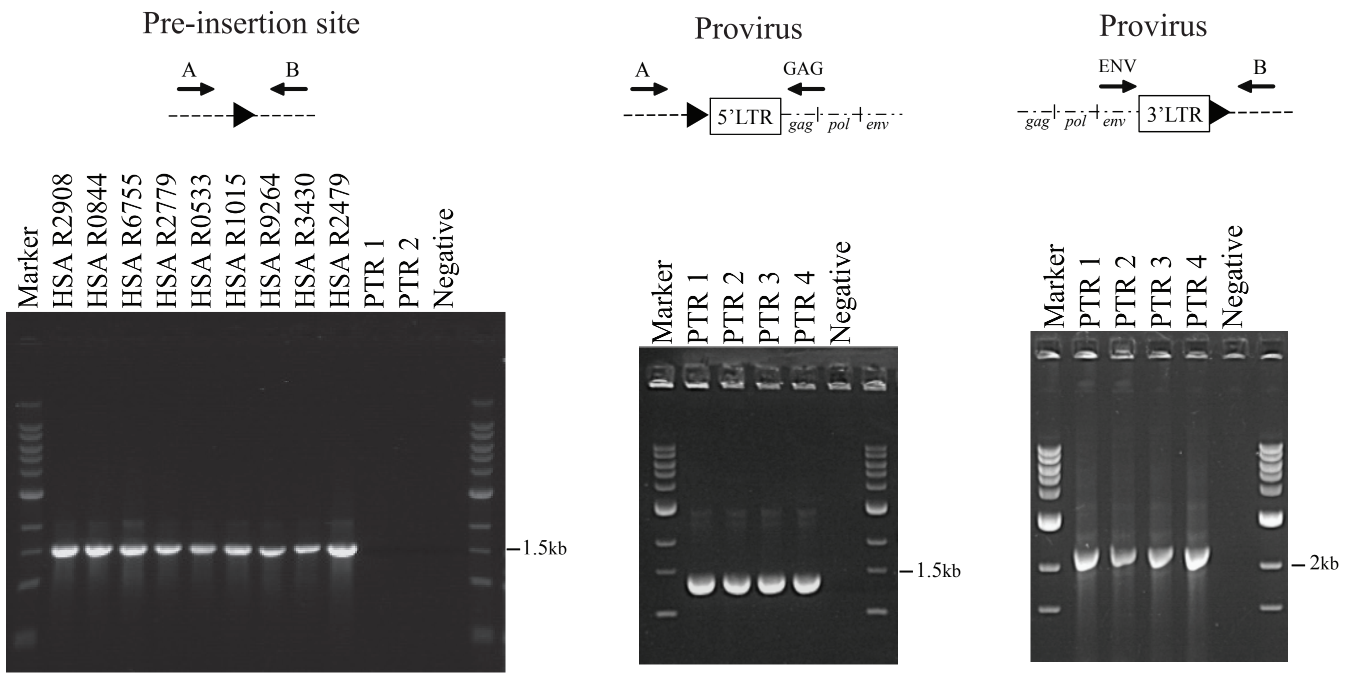

B Pan8q

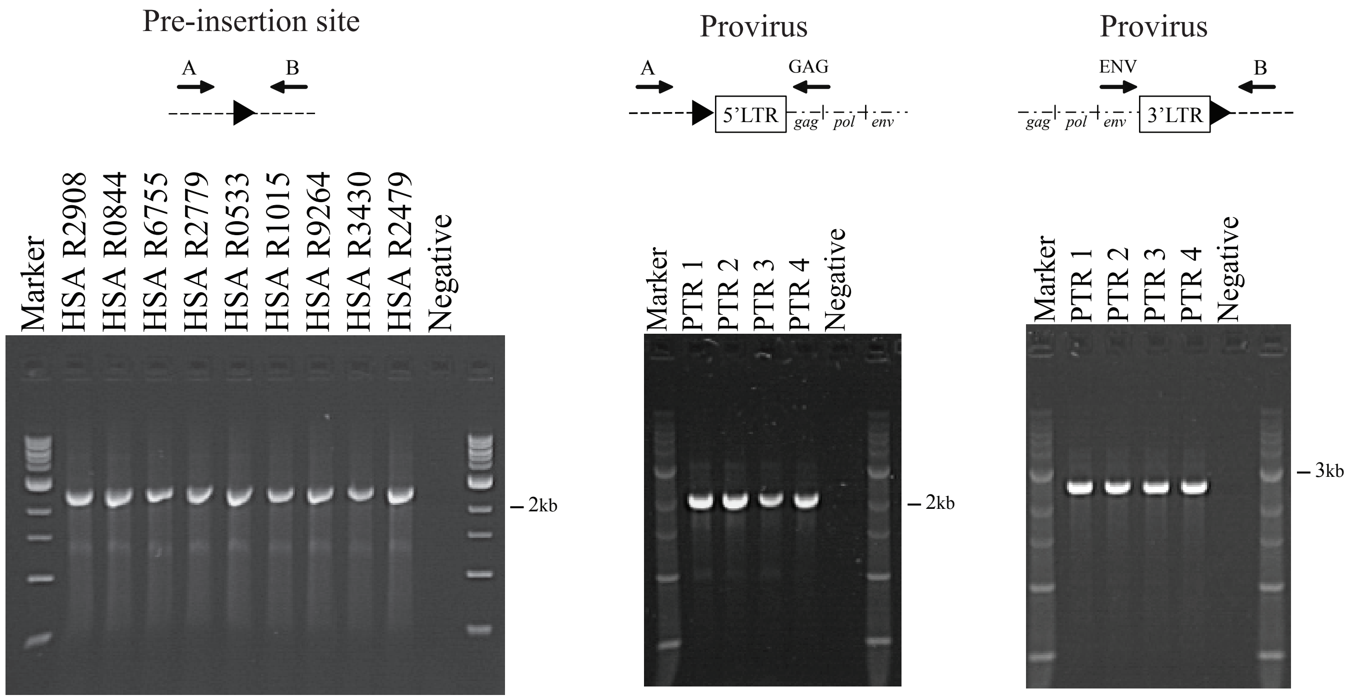

Supplement: Additional file 8: — Agarose gel images of PCR genotyping for HERV-K(HML-2) proviruses in human blood and chimpanzee panel DNAs. (A) Pan2Ap. (B) Pan8q. The locus specific primers A + B amplify the pre-insertion site and solo LTR. Primer combination A + GAG amplifies the 5′ LTR of a full-length provirus and ENV + B the 3′ LTR. Numbers on the left correspond to the co-migrating DNA size marker (kb). The identity of each band was confirmed by DNA sequencing. [file 12977_2015_162_MOESM8_ESM.pdf]
