# Supplementary material for: Genome-wide amplification of proviral sequences reveals new polymorphic HERV-K(HML-2) proviruses in humans and chimpanzees that are absent from genome assemblies
Source: Retrovirology. 2015 Apr 28;12:35. doi: 10.1186/s12977-015-0162-8 (PMC4422153; doi:10.1186/s12977-015-0162-8)

Additional File 9

Agarose gel images of PCR genotyping for dimorphic HERV-K(HML-2) proviruses in the ECAAC human ethnic diversity panel (Samples A1 to H12)

A

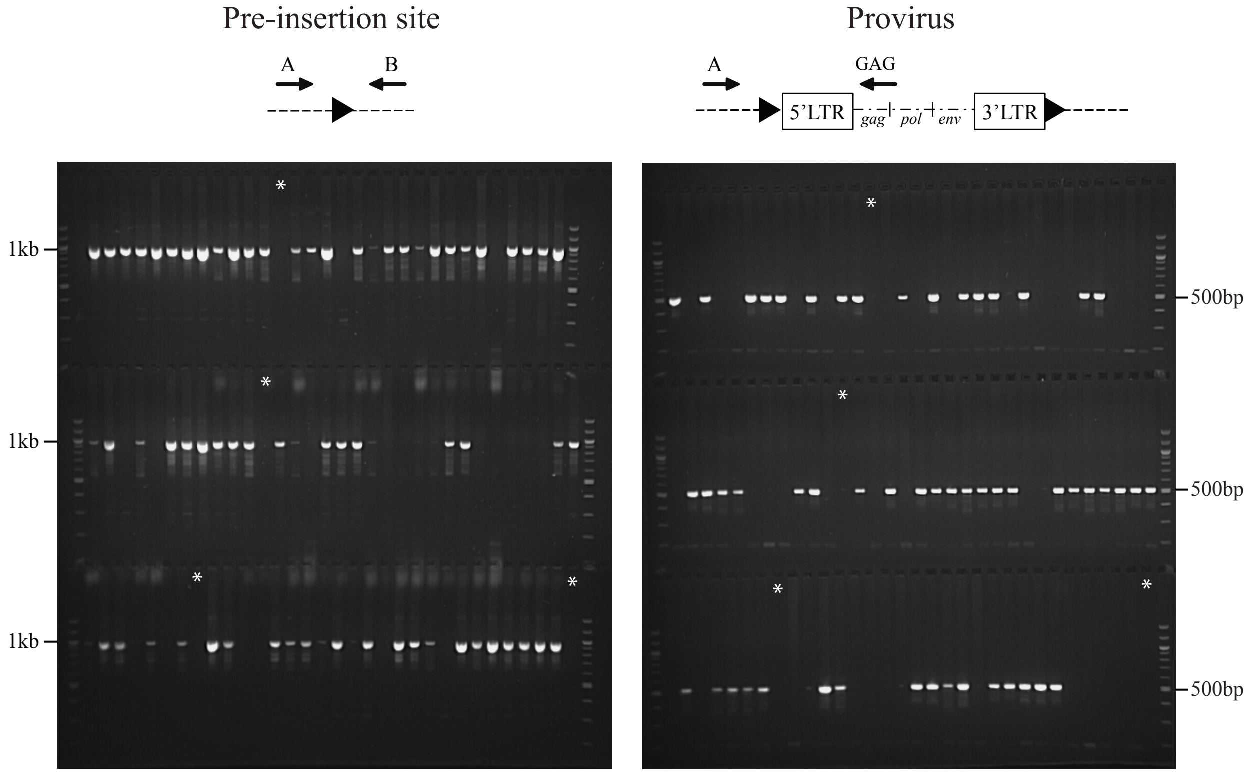

B

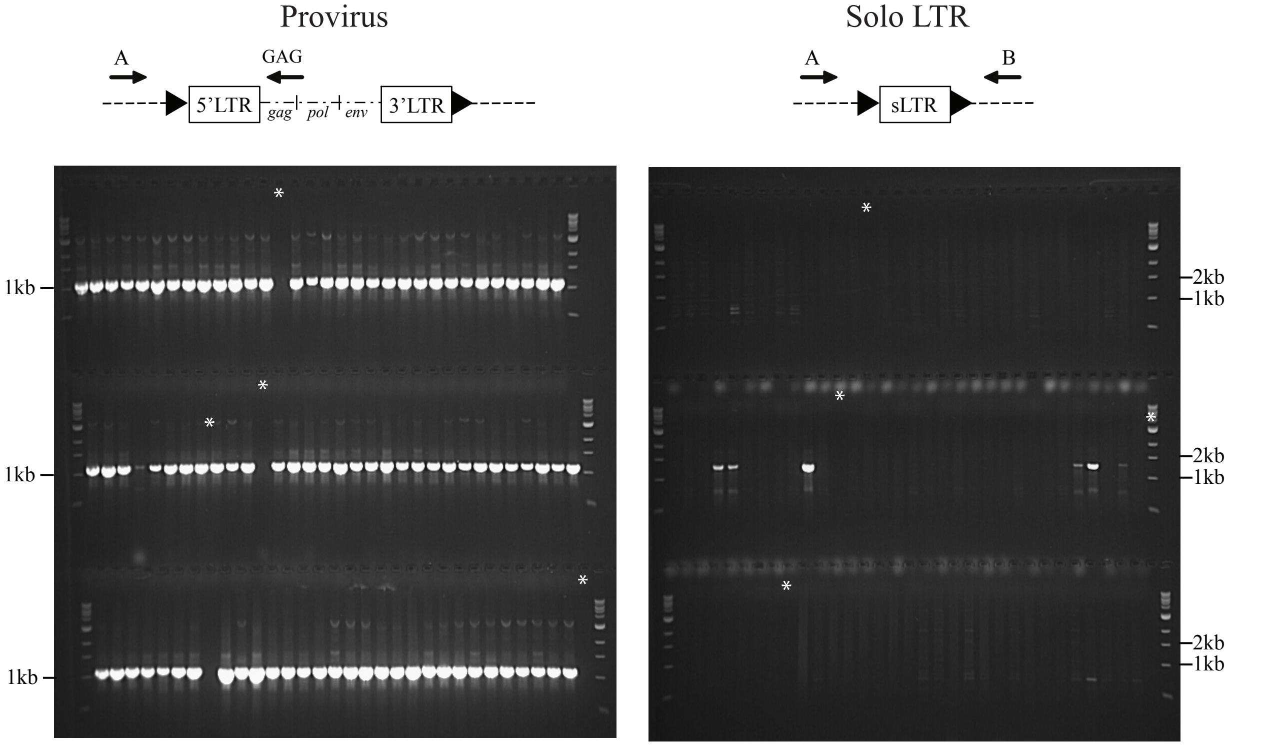

Supplement: Additional file 9: — Agarose gel images of PCR genotyping for dimorphic HERV-K(HML-2) proviruses in the ECAAC human ethnic diversity panel (Samples A1 to H12). (A) 19p12c. The gel on the right shows amplification for the pre-insertion site using primer combination A + B. The left gel shows amplification with primers A + GAG for the provirus. There are four no DNA blanks within the ECAAC panel (F2, D6, H9 and H12) which have, as anticipated, not generated GAPS amplicons, these are denoted by a *. (B) 1p31.1a. The gel on the right shows amplification for the provirus using primer combination A + GAG. The left gel shows amplification for the Solo LTR using primers A + B. There are four no DNA blanks within the ECAAC human ethnic diversity panel (F2, D6, H9 and H12) which have, as anticipated, not generated GAPS amplicons, these are denoted by a *. [file 12977_2015_162_MOESM9_ESM.pdf]
