# Supplementary material for: Genome-wide amplification of proviral sequences reveals new polymorphic HERV-K(HML-2) proviruses in humans and chimpanzees that are absent from genome assemblies
Source: Retrovirology. 2015 Apr 28;12:35. doi: 10.1186/s12977-015-0162-8 (PMC4422153; doi:10.1186/s12977-015-0162-8)

Additional File 10

Genomic Location, gene density and local recombination rate surrounding the HERV-K(HML-2) proviruses 1p31.1a and 1p31.1b

A

UCSC Genome Browser on Human Feb. 2009 (GRCh37/hg19)
chr1:72594981-74595948 (2,000,968 bp)

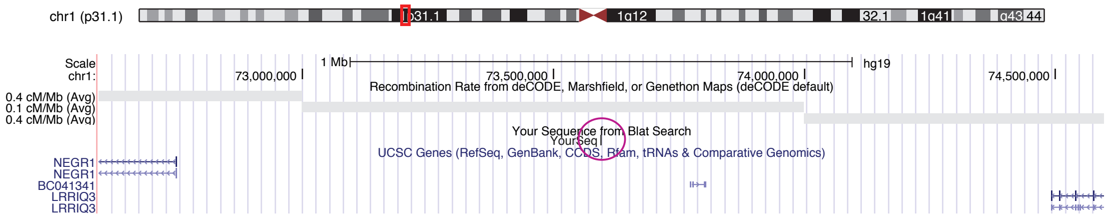

B

UCSC Genome Browser on Human Feb. 2009 (GRCh37/hg19)
chr1:74,842,771-76,849,143 (2,006,373 bp)

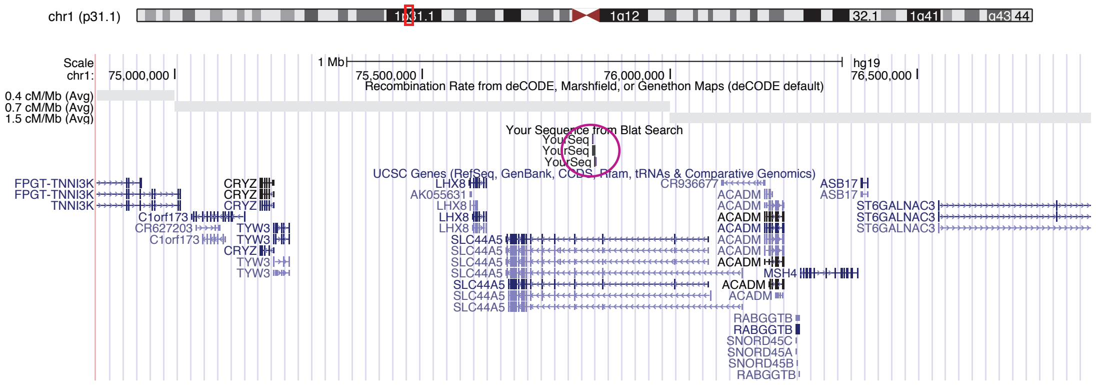

Supplement: Additional file 10: — Genomic Location, gene density and local recombination rate surrounding the HERV-K(HML-2) proviruses 1p31.1a and 1p31.1b. (A) UCSC Genome Browser screen capture of the ancient 1p31.1a locus. (B) UCSC Genome Browser screen capture of the human specific 1p31.1b locus. The human specific HERV-K (HML-2) 1p31.1b provirus is also variable for a solo LTR and a provirus and is located ~ 2Mb downstream of the ancient HERV-K (HML-2) 1p31.1a locus. The locations of the loci are highlighted in red circles. The figures show the genes and local recombination rates 1Mb upstream and 1Mb downstream of each of the loci. [file 12977_2015_162_MOESM10_ESM.pdf]
