# Supplementary material for: Genome-wide amplification of proviral sequences reveals new polymorphic HERV-K(HML-2) proviruses in humans and chimpanzees that are absent from genome assemblies
Source: Retrovirology. 2015 Apr 28;12:35. doi: 10.1186/s12977-015-0162-8 (PMC4422153; doi:10.1186/s12977-015-0162-8)
Supplement: Additional file 11: — Primers. [file 12977_2015_162_MOESM11_ESM.pdf]

Additional File 11

Primers

Primer	Sequence
RBMSL2	GTGGCGGCCAGTATTCGTAGGAGGGCGCGTAGCATAGAACG
RBD3	TACGTTCTATGCTAC
RBX4	GTGGCGGCCAGTATTC
RBY1	GAGGGCGCGTAGCATAGAAC
CMKGAG	GGATCTCTCGTCGACTTGTC
CMKENV	CAGGTGTACCCAACAGCTC
CMK5LTR	AATGGAGTCTCCTATGTCTACT
CMK3LTR	GATCCTCCATATGCTGAACG
CMK1p311A	ACCAGTGACTGCCTCAGGTT
CMK1p311B	CACACAGCTTGAGGTTCCAG
CMKPAN2APA	TCCTGCTCTTTTGCTTCTCC
CMKPAN2APB	CACCCCTCCTTCATGAGAAA
CMKPAN8QA	ATTGAAAGAAGTATAAGGTGTAAGTAAGG
CMKPAN8QB	CATATGGGTGTTCCCTTATATGT
CMK19P12A	TGTAATGGCTTGTTTTGTCTCTG
CMK19P12B	GCATAATCATAATGGTGCAGAAAA
CMK3LTRAS	CGTTCAGCATATGGAGGATC
CMKF0001	TGTGGGGAAAAGCAAGAGAG
CMKF0520	GAATGTCTCGGTATAAAACCCG
