# Supplementary material for: Genome-wide amplification of proviral sequences reveals new polymorphic HERV-K(HML-2) proviruses in humans and chimpanzees that are absent from genome assemblies
Source: Retrovirology. 2015 Apr 28;12:35. doi: 10.1186/s12977-015-0162-8 (PMC4422153; doi:10.1186/s12977-015-0162-8)
Supplement: Additional file 12: — PCR Genotyping conditions. [file 12977_2015_162_MOESM12_ESM.pdf]

Additional File 12

PCR Genotyping conditions

Locus variant	Primers	Cycle conditions
19p12c Pre-insertion site	CMK19p12A + CMK19p12B	96-1;30[96-30s;60-30s;72-90s]72-2min
19p12c Provirus 5'LTR	CMK19p12A + CMKGAG	96-1;30[96-30s;60-30s;72-90s]72-2min
19p12c Provirus 3'LTR	CMK19p12B + CMKENV	96-1;30[96-30s;60-30s;72-90s]72-2min
1p31.1a Provirus 5'LTR	CMK1p311A + CMK1p311 GAG	96-1;30[96-30s;60-30s;72-90s]72-2min
1p31.1a Provirus 3'LTR	CMK1p311B + CMKENV	96-1;30[96-30s;60-30s;72-90s]72-2min
1p31.1a Solo LTR	CMK1p311A + CMK1p311B	96-1;30[96-30s;60-30s;72-90s]72-2min
Pan2Ap Pre-insertion site	CMKPan2ApA + CMK Pan2ApB	96-1;30[96-30s;60-30s;72-90s]72-2min
Pan2Ap Provirus 5'LTR	CMKPan2ApA + CMKGAG	96-1;30[96-30s;60-30s;72-90s]72-2min
Pan2Ap Provirus 3'LTR	CMKPan2ApB + CMKENV	96-1;30[96-30s;60-30s;72-90s]72-2min
Pan8q Pre-insertion site	CMKPan8qA + CMKPan8qB	96-1;30[96-30s;58-30s;72-90s]72-2.5min
Pan8q Provirus 5'LTR	CMKPan8qA + CMKGAG	96-1;30[96-30s;58-30s;72-90s]72-2.5min
Pan8q Provirus 3'LTR	CMKPan8qB + CMKENV	96-1;30[96-30s;58-30s;72-90s]72-2.5min
